# The viral and host genomic landscape of human T-cell leukemia virus type I in Peru

**DOI:** 10.1186/s12985-026-03148-8

**Published:** 2026-04-06

**Authors:** Daniel Enriquez-Vera, Jiazhou Li, Jorge Nakazaki-Aza, Kosuke Mochida, Yutaka Suzuki, Eduardo Gotuzzo, Martín Montes, Kazuhiro Morishita, Shingo Nakahata

**Affiliations:** 1 Division of HTLV-1/ATL Carcinogenesis and Therapeutics, Joint Research Center for Human Retrovirus Infection, Kagoshima University, 8-35-1 Sakuragaoka, Kagoshima-shi, Kagoshima, 890-8544 Japan; 2 Facultad de Medicina, Instituto de Medicina Tropical ‘Alexander von Humboldt’, Universidad Peruana Cayetano Heredia, Honorio Delgado 430, San Martin de Porres, Lima, 15102 Peru; 3 Department of Dermatology, Department of Medical Sciences, University of Miyazaki, 5200 Kihara, Kiyotake, Miyazaki, 889-1692 Japan; 4 Laboratory of Systems Genomics, Department of Computational Biology and Medical Sciences, Graduate School of Frontier Sciences, The University of Tokyo, Kashiwa, Chiba, 277-8561 Japan; 5 Division of Pediatrics, Department of Developmental and Urological-Reproductive Medicine, Faculty of Medicine, University of Miyazaki, 5200 Kihara, Kiyotake, Miyazaki, 889-1692 Japan; 6 Division of Tumor and Cellular Biochemistry, Department of Medical Sciences, University of Miyazaki, 5200 Kihara, Kiyotake, Miyazaki, 889-1692 Japan

**Keywords:** Human T-lymphotropic virus 1, Leukemia-Lymphoma, Adult T-Cell, Clonal Hematopoiesis, Health Disparities, Neglected Diseases, Latin America

## Abstract

**Background:**

The human T-cell leukemia virus type 1 (HTLV-1) is a neglected oncogenic retrovirus responsible for adult T-cell leukemia/lymphoma (ATLL) and autoimmune diseases that disproportionately affects marginalized populations worldwide. Peru reports the highest global ATLL incidence, yet comprehensive genomic studies remain limited. We aimed to characterize the virological, immunological, and host genetic landscape of HTLV-1 infection to identify population-specific high-risk features.

**Methods:**

We conducted a prospective cohort study (2017–2018) of 67 HTLV-1-infected individuals from Lima, Peru, using an integrated high-throughput genomic approach. This included whole-genome HTLV-1 sequencing, targeted ultra-deep sequencing of 280 hematological malignancy-associated genes, high-resolution HLA typing, GATK-based variant calling, and comprehensive clinical follow-up over 7 years. Phylogenomic analyses were performed using maximum likelihood and Bayesian approaches.

**Results:**

The cohort exhibited exceptionally high-risk characteristics with a median proviral load (PVL) of 4.5 ± 3.8, and 13.4% cumulative crude mortality over 7 years. Phylogenomic analysis revealed 96.8% of isolates belonged to the Transcontinental subtype, with most clustering in a newly identified Andean-Amazonian subgroup. HLA-I analysis demonstrated unique population-specific allele distributions with significantly reduced evolutionary divergence compared to Japanese cohorts (HLA-HED: 5.34 vs. 6.87, *p* = 0.0002), potentially differences in mechanisms of viral immune control. Ultra-deep sequencing identified early clonal hematopoiesis with prevalent mutations in cancer-associated genes including KMT2D (55%), NOTCH1 (49%), and TP53 (27%). Mutation burden correlated significantly with proviral load (*r* = 0.34, *p* = 0.003), and longitudinal analysis revealed progressive genomic instability with more than two-fold increase in mutations over 3 years (7.25 vs. 19.5 mutations per patient, *p* = 0.01). High PVL (> 4%) was the only independent predictor of crude mortality (OR: 1.07; 95% CI: 1.01–1.15; *p* = 0.033). Contrary to previous reports, *Strongyloides* coinfection was not associated with disease progression.

**Conclusions:**

This first comprehensive genomic characterization of HTLV-1 in South America reveals population-specific viral evolution, reduced HLA diversity, and evidence of early oncogenic transformation events. The exceptionally high proviral loads and unique mutational landscape provide novel insights into HTLV-1 pathogenesis and support the development of population-tailored risk stratification approaches. These findings emphasize the urgent need for expanded genomic surveillance and targeted interventions in underrepresented populations bearing disproportionate HTLV-1 burden.

**Supplementary Information:**

The online version contains supplementary material available at 10.1186/s12985-026-03148-8.

## Introduction

The human T-cell leukemia virus type 1 (HTLV-1) is a neglected oncogenic retrovirus that disproportionately affects marginalized and isolated populations worldwide. Following several decades of chronic infection, HTLV-1 directly causes an aggressive form of T-cell lymphoma (adult T-cell leukemia/lymphoma, ATLL) and several autoimmune disorders, most notably HTLV-1-associated myelopathy/tropical spastic paraparesis (HAM/TSP) [1]. Even seemingly asymptomatic carriers (ACs) face significantly elevated mortality risk from indirect complications, independent of malignant transformation [2, 3]. While transmission has been successfully controlled in some developed regions, HTLV-1 remains largely neglected across most of the global south, where it continues to cause substantial morbidity and mortality [4, 5]. The burden is particularly pronounced in rural populations with limited genetic admixture and restricted access to healthcare [6–9]. 

Efforts to identify high-risk ACs have been focused on three key determinants: viral, host, and environmental factors. Among viral factors, current HTLV-1 classification systems (e.g., Cosmopolitan [a–d], Japanese, West African) are based on the long terminal repeat (LTR) and *env* gene diversity, which correlates with geographical distribution but lacks clear associations with clinical outcomes. Functional genomic differences remain controversial, though Tax A (Transcontinental) variants demonstrate trends toward increased HAM/TSP incidence, reduced survival among ATLL cases, and diminished cytotoxic T-lymphocyte activity compared to Tax B (Japanese) variants [10–13]. Clinically, HTLV-1 proviral load (PVL) and oligoclonality index (OCI) represent the most widely adopted viral prognostic biomarkers, while emerging evidence suggests that defective proviruses may facilitate immune evasion during ATLL progression [14, 15]. However, these findings have been predominantly validated in Japanese populations, limiting their broader applicability.

Host genetic factors appear central to determining why only a small proportion (less than 10%) of ACs eventually develop ATLL or HAM/TSP. Early epidemiological observations—including familial clustering and disproportionate disease prevalence in specific ethnic populations—strongly implicate genetic susceptibility factors [16–18]. Supporting this hypothesis, a recent meta-analysis identified specific human leukocyte antigen (HLA) alleles associated with either protection against HTLV-1 infection or modulation of disease progression [19]. Furthermore, genomic and transcriptomic studies have revealed distinct age- and population-specific differences between Western and Japanese cohorts, highlighting how host-pathogen interactions and genetic background influence HTLV-1-related disease susceptibility and severity [20, 21]. 

Environmental factors, particularly *Strongyloides* coinfection, have also been linked to ATLL progression, though the underlying mechanisms remain poorly understood. In that sense, South America (SA) exhibits high prevalence rates for both HTLV-1 and *Strongyloides* infection. Specifically, Peru represents a population of scientific interest since it reported the highest ATLL incidence globally and the largest proportion of indigenous ancestry in the South American region [22, 23]. Despite this epidemiological significance, comprehensive genomic studies of HTLV-1-infected individuals in Peru remain limited. In consequence, we aimed to characterize the virological, immunological, and host genetic landscape of HTLV-1 infection using an integrated high-throughput genomic approach in a well-characterized Peruvian cohort, with the purpose of identifying high-risk features and advancing our understanding of HTLV-1 pathogenesis in underrepresented populations.

## Methods

### Population study, HTLV-1 confirmation and PVL measurement

Blood samples were collected from participants in the prospective HTLV-1 cohort at Instituto de Medicina Tropical Alexander von Humboldt (Universidad Peruana Cayetano Heredia, Lima, Peru) between 2017 and 2018. All participants had previously been screened for HTLV-1 and confirmed positive by enzyme-linked immunosorbent assay (ELISA), Western-blot, and PCR-based testing at Universidad Peruana Cayetano Heredia. Participants were included regardless of their proviral load. Vital status follow-up was based on medical records and the national vital status system (Registro Nacional de Identidad y Estado Civil, RENIEC). Mortality was confirmed when both registries consistently showed a death entry. The crude mortality rate included malignant and non-malignant causes of death, calculated using the total population but excluding cases lost to follow-up.

DNA was extracted from peripheral blood mononuclear cells (PBMCs) using the Ficoll gradient technique (Genomic DNA Isolation Kit, Norgen Biotek Corp., Thorold, ON, Canada). DNA purity was assessed using the Qubit dsDNA HS Assay Kit (Thermo Fisher Scientific, Waltham, MA, USA). HTLV-1 PVL was quantified using real-time quantitative PCR (qPCR) targeting the HTLV-1 *pX* gene and *β-globin* gene as an internal control. The *pX* primers were: forward 5’-GGGATTACCGGCTCCATGTC-3’ and reverse 5’-TCAAGGCCTCGTCTGTTCTG-3’, amplifying an 80 base-pair product. The *β-globin* primers were: forward 5’-GCAAGGTGAACGTGGATG-3’ and reverse 5’-TAAGGGTGGGAAAATAGACC-3’. The qPCR reactions were performed using the Applied Biosystems StepOnePlus™ Real-Time PCR System with THUNDERBIRD^®^ SYBR^®^ qPCR Mix (201-T, TOYOBO). The thermal cycling conditions included initial pre-denaturation at 95 °C for 60 s, followed by 30 cycles of denaturation at 95 °C for 15 s, annealing at 59 °C for 15 s, and extension at 72 °C for 45 s. Each sample was analyzed in triplicate, and standard curves were generated using serial dilutions of high-quality DNA extracted from the MT-2 cell lines. The HTLV-1 proviral load was calculated as: PVL (%) = [(HTLV-1 copy number)/(β-globin copy number/2)] × 100. The calculation assumes diploid human genome (2 copies of β-globin per cell) and uses MT-2 (JCRB Cell Bank) containing one integrated HTLV-1 proviral copy and ATL-48T (RIKEN BRC Cell Bank) containing two integrated HTLV-1 proviral copies [24] as the reference standard for quantification. Results were expressed as the percentage of HTLV-1 proviral copies per 100 PBMCs. Samples with coefficient of variation > 15% between triplicates were repeated. The detection limit was established at 0.001% based on the lowest dilution of positive controls showing consistent amplification.

### Next-Generation Sequencing and Human Targeted Sequencing

Next-Generation Sequencing (NGS) Library preparation was performed using the SureSelect XT HS Kit (Agilent Technologies, Santa Clara CA, USA) following the manufacturer’s protocol. Target sequencing for 280 human genes and HTLV-1 genome was performed as previously described (panel-based ultra-deep sequencing) [25]. Sequencing was conducted on Illumina Hiseq3000 sequencer with 100base-pair paired-end reads. Raw reads were first analyzed for quality and adapter removal using FastQC (0.12.1), Fastp (0.23.4), and multiQC (1.27.1). The sequenced data were aligned to the human reference genome hg38 plus HTLV-1 (J02029.1) as an additional chromosome, using BWA-MEM2 (2.2.1) in a customized bash script available in the paper’s repository. The clonality of HTLV-1-infected cells was assessed using previously established methods, including the calculation of the oligoclonality index (OCI) based on Gini’s index. The OCI was derived from consistently chimeric well-paired reads and compared across multiple algorithms, including Virusbreakend (2.13.2) and Viruscan (1.0) [26]. 

Post-alignment processing was performed following the GATK (4.6) best practices pipeline, including marking or removing duplicates with Picard, and base quality recalibration. Variant calling was conducted using GATK’s Mutect2, followed by annotation with Funcotator, leveraging standard databases such as dbSNP, HUGO, and COSMIC for functional interpretation. A pool of normals (PON) derived from the 1000 Genomes Project was used to filter out germline variants and sequencing artifacts, ensuring high specificity for somatic mutation detection. Candidate mutations were filtered based on the following criteria: (1) at least 5 consistent paired reads supporting the variant, (2) a variant allele frequency (VAF) ≥ 0.01, and (3) a minimum read depth of 200. In addition, variants with a frequency larger than 35% were excluded from the analyses. This rigorous filtering approach ensures the identification of high-confidence somatic mutations while minimizing false positives. Quality control of the filtered variants was evaluated using IVG. All pipelines with software citations have been detailed in Supplementary Methods 1 and the paper repository (https://github.com/deve105/htlv_paper_2025).

### HTLV-1 Genome Sequencing and Phylogenetic Analysis

Consensus HTLV-1 sequences were generated using a standardized bioinformatics pipeline. Raw sequencing reads were quality-filtered and mapped to the HTLV-1 reference genome (J02029.1) using BWA-MEM2. Post-mapping processing was performed using Samtools with stringent quality parameters (minimum base quality 20, frequency threshold 0.8), followed by consensus sequence generation using IVAR (1.4). Quality control measures included removal of low-quality sequences, PCR duplicates, and sequences with < 60% pairwise identity to the reference genome. Four samples (IRID011, IRID038, IRID040, IRID067) were excluded due to insufficient sequence quality or coverage (< 6,000 bp after trimming). A comprehensive reference dataset of curated HTLV-1 complete genomes was retrieved from NCBI GenBank (accessed January 2024), including sequences from global collections representing major geographical regions and subtypes. All sequences were trimmed to remove variable terminal regions (22 nucleotides from 5’LTR and 13 nucleotides from 3’LTR) to ensure consistent alignment boundaries. Multiple sequence alignment (MSA) was performed using MAFFT (7.49) with the L-INS-i algorithm, optimized for sequences with conserved domains and local homology. Alignment quality was assessed through visual inspection and automated gap analysis, with sequences showing < 60% pairwise identity excluded from downstream analyses. Optimal evolutionary models were selected using ModelFinder implemented in IQ-TREE (3.0.1), with PartitionFinder used to determine the best-fitting three-partition scheme (LTR + env + whole genome) based on corrected Akaike Information Criterion (cAIC) and Bayesian Information Criterion (BIC). Detailed information on partitioning and model selection is presented in Supplementary Methods 2. Maximum likelihood (ML) phylogenetic reconstruction was performed using RAxML-NG (1.2.2) with 5,000 bootstrap replicates to assess node support. Convergence was verified through multiple independent runs and examination of likelihood scores. Cluster analysis and genetic diversity patterns were explored using multiple complementary approaches: ML phylogenetics, Bayesian inference, Discriminant Analysis of Principal Components (DAPC), and k-means clustering. Bayesian phylodynamic and phylogeographic analyses were conducted using BEAST (2.7) to estimate divergence times and ancestral geographic states. For tree visualization and comparison, identical sequences were collapsed to reduce computational burden while maintaining phylogenetic signal.

Recombination analyses.

Recombination events were systematically evaluated using a multi-step approach to ensure robust detection and minimize false positives. Initial screening was performed using the phi-test for overall recombination signal detection, followed by comprehensive exploratory analysis using seven independent algorithms implemented in RDP (5.8.1): RDP, GENECONV, Bootscan, MaxChi, SisCan, 3Seq, and Chimaera. Each method employs distinct statistical approaches to identify potential recombination breakpoints and assess significance. Potential recombination events were considered significant when detected by at least four independent methods with Bonferroni-corrected p-values ≤ 0.05. This conservative threshold was chosen to minimize false positives while maintaining sensitivity for genuine recombination events. Sequences showing evidence of recombination were subsequently analyzed using the Genetic Algorithm for Recombination Detection (GARD) implemented in HyPhy (2.5) to confirm breakpoint locations. Following recombination confirmation, affected sequences were excluded from downstream phylogenetic analyses to prevent artifacts in tree topology and divergence time estimation. The final dataset comprised only sequences without evidence of recombination, ensuring phylogenetic signal integrity for subsequent molecular evolutionary analyses. Quality control measures included visual inspection of recombination plots, assessment of breakpoint confidence intervals, and evaluation of phylogenetic incongruence patterns. All recombination analysis parameters and results are documented in Supplementary Tables 1 and Supplementary Fig. 1, with detailed breakpoint coordinates and supporting statistics provided for transparency and reproducibility.

### HLA Analysis

Following reads extraction from the chromosome 6, coverage and quality score analyses were performed for all HLA regions. High-resolution HLA typing was carried out using OptiType (Version 1.3.5, 2-field resolution for HLA-I) and HLA-LA (Version 1.0.4, 3-field resolution for HLA-I/II). Allotypes failing quality thresholds (score < 0.8 or average coverage < 20) were discarded. Results from both bioinformatics tools were pairwise compared, and, considering the low average coverage for HLA-II, only HLA-I was considered for further analysis. The concordances and Cohen’s Kappa between HLA-LA and Optitype were 93%, 85%, and 100% for HLA-A, HLA-B, and HLA-C, respectively. The HLA-LA pipeline was selected for further analyses. Hardy-Weinberg Equilibrium (HWE) was tested for each locus using the exact test implemented on the pegas package with 1000 replicates with a significance threshold of α = 0.05. Pairwise FST was estimated using Weir & Cockerham’s method with 10,000 bootstrap replicates implemented on the package hierfstat. Significant differentiation was inferred when 95% CIs excluded zero. HLA class I allele frequencies were calculated after reduction to 2-field resolution using the *reduceHlaCalls* function from the package midasHLA. Frequencies for the Peruvian population were obtained using the function *getHlaFrequencies* and compared to the USA National Marrow Donor Program (NMDP) populations that were retrieved from Allele Frequency Net Database (http://www.allelefrequencies.net/). The HLA-I associations analyses at allele and aminoacid levels were performed using the dominant inheritance genetic model, Bonferrini correction, and filtering for at least 10% frequency of HLA alleles. The event of interest for association studies was confirmed mortality. The HLA-I associations analyses were explored in Rstudio using the package midasHLA. Evolutionary divergence of the HLA-I genes (HED) was evaluated using the Grantham distance at HLA-A, HLA-B, and HLA-C. Grantham distance takes into account composition, polarity, and volume. HED was calculated using a custom Python script. The HLA NGS data for the HTLV-1/ATLL cohort from Miyazaki, Japan, were derived from a previous study, in which the data were obtained using the same methodology as employed in the present study [27]. 

## Results

### 1. Clinical and epidemiological features of the Peruvian HTLV-1 cohort

A total of 67 patients with confirmed HTLV-1 infection were enrolled in the study, including 10 HAM/TSP patients and 57 ACs. Detailed demographic and epidemiological characteristics are presented in Table 1. The overall lifetime *Strongyloides* infection (LSI) rate was 56.7%, with a higher prevalence among males. Over the 7-year follow-up, the progression rate to ATL was 5.3% (2 in ACs cases, and 1 in HAM/TSP cases). No association was observed between LSI and ATLL development. Interestingly, HAM/TSP patients had a lower frequency of LSI compared to ACs. The PVL in this cohort was high, with a median of 4.5 ± 3.8, indicating that it represents a high-risk carrier population [15]. Furthermore, the median PVL was higher in the LSI group (5%) compared to the non-LSI group (3.3%), but this difference did not reach statistical significance. Notably, high-risk HTLV-1 infection (PVL > 4%) was observed to be more frequent in the LSI group than in the non-LSI group (68 vs. 48%, respectively). Generalized linear models, including both beta regression and robust regression, were applied to evaluate predictors of PVL. Neither demographic (age, sex, birthplace) nor LSI were significantly associated with higher PVL in either model (all *p* > 0.05, Supplementary Table 2). A total of 9 confirmed deaths (cumulative crude mortality risk 13.4%, 95% Confidence Interval [CI]: 6.3%–24.0%) were observed during the 7-year follow-up, with 3 cases related to malignant transformation and 6 related to infectious complications (during COVID-19 pandemic). Additionally, 8 cases were lost to follow-up, and their vital status could not be confirmed. In a multivariable regression model adjusted for age, sex, PVL, and *Strongyloides* status, PVL was the only independent factor significantly associated with increased crude mortality (adjusted OR: 1.07; 95% CI: 1.03–1.79; *p* = 0.047; Supplementary Table 3).

**Table 1:** Demographic and Clinical Characteristics of HTLV-1-Infected Individuals by LSI Status

Variable	Strongyloides (-) *N* = 29 (43.3%)^*1*^	Strongyloides (+) *N* = 38 (56.7%)^*1*^	*p*-value^2^	Total^1^
**Sex**			0.039	
Female	21 (72)	18 (47)		39 (58)
Male	8 (28)	20 (53)		28 (42)
**Age (years)**	49.5 (38.5, 55.0)	49.5 (39.0, 54.0)	> 0.9	49.5 (39.0, 54.0)
**Birthplace**			0.4	
Amazonian region	0 (0)	2 (6.1)		2 (3.6)
Andean region	8 (35)	14 (42)		22 (39)
Coastal region	15 (65)	17 (52)		32 (57)
**Proviral Load (PVL**,**%)**	3.3 (1.1, 6.4)	5.0 (3.2, 6.9)	0.11	4.5 (2.5, 6.9)
**PVL-Risk (≥ 4%)**			0.10	
High-Risk	14 (48)	26 (68)		40 (60)
Low-Risk	15 (52)	12 (32)		27 (40)
**Oligoclonality Index (OCI)**	0.2 (0.1, 0.2)	0.2 (0.1, 0.2)	0.9	0.2 (0.1, 0.2)
**7-year Disease Outcome**			< 0.001	
Asymptomatic carriers	18 (62)	36 (95)		54 (81)
ATLL	2 (6.9)	1 (2.6)		3 (4.5)
HAM/TSP	9 (31)	1 (2.6)		10 (15)
**7-year Status**			0.5	
Alive	19 (79)	31 (89)		50 (85)
Death	5 (21)	4 (11)		9 (15)
Lost-to Follow-up	5 (17)	3 (8)	0.3	8 (14)

^1^n (%); Median (Q1, Q3)

^2^Pearson's Chi-squared test; Wilcoxon rank sum test; Fisher's exact test; Wilcoxon rank sum exact test

### 2. HTLV-1 genomics and phylogenomics features

**Fig. 1:**
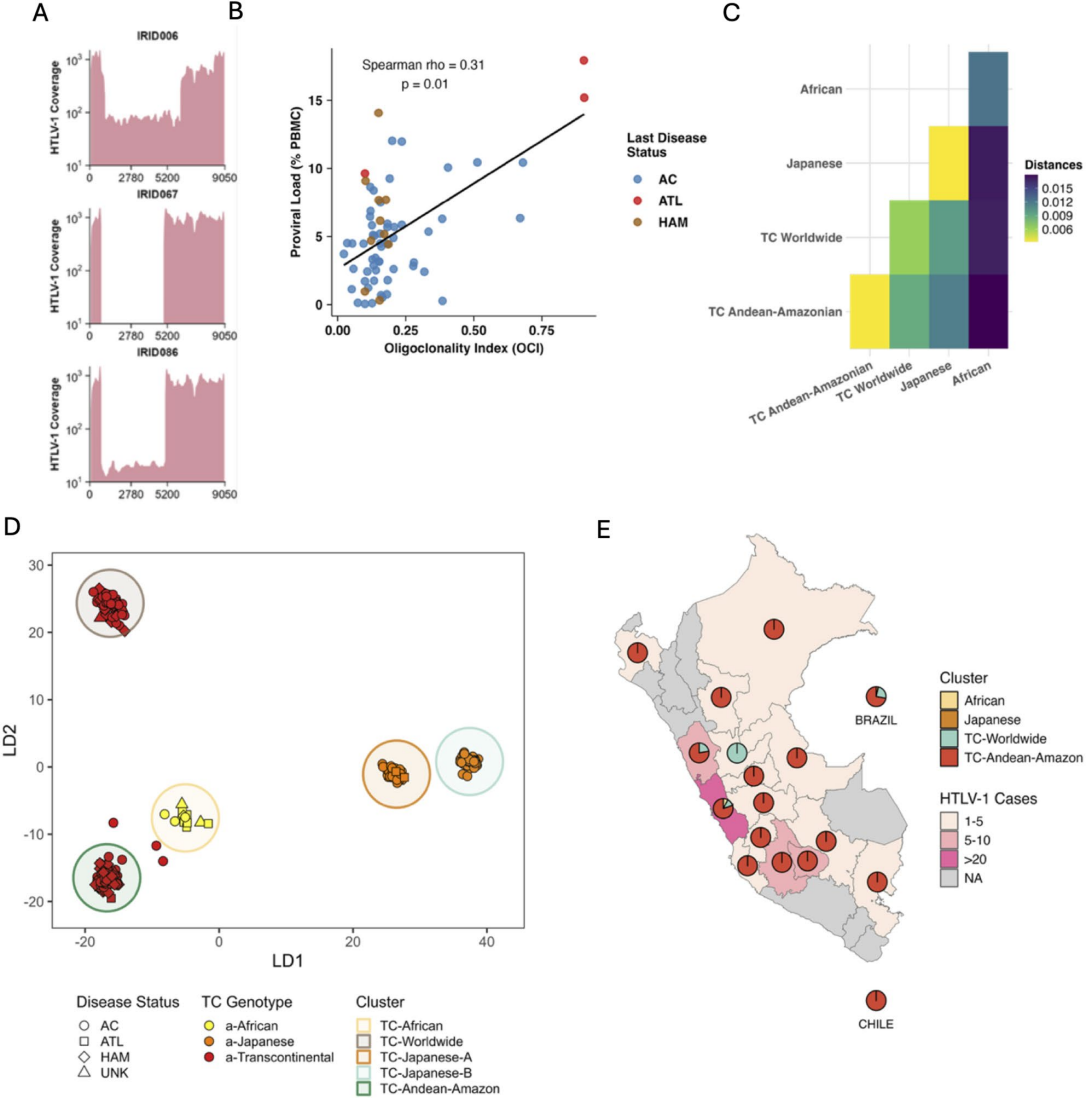
HTLV-1 genomics features of the Peruvian cohort. **A**) Identification of defective Type II HTLV-1 variants (5’LTR deletion) within the cohort, characterized by a reduction of at least 10% in total genome coverage relative to the J02029.1 reference genome (expressed on a log scale and using mosdepth [v3.1]). These three identified events were associated with progression to ATLL transformation or infection-related mortality. **B**) Correlation analysis between Proviral Load (PVL) and Oligoclonality Index (OCI; calculated using Gini’s Index) was evaluated using Spearman’s rank correlation. Clinical outcomes were color-coded: Asymptomatic Carriers (AC, blue), HAM/TSP (brown), and ATLL (red). The black line represents the linear regression fit, illustrating the relationship between clonal expansion and viral load. **C**) Nucleotide distances between clusters within the Cosmopolitan clade were calculated using the Kimura 2-parameter (K80) evolutionary model. This analysis provides insights into genetic divergence and sequence variability across HTLV-1 samples. The analysis encompasses all sequences clustered under African subtypes (a-NA, a-WA, a-Sen, a-Rec, a-Per or also known as Black Peruvians), the Japanese subtype (a-Jpn), and a-TC, which is further subdivided into a-TC-Andean-Amazonian and a-TC-worldwide. **D**) Unsupervised clustering performed using dudiPCA, excluding samples from Australo-Melanesian origin (HTLV-1c), revealing two well-defined clusters within the Transcontinental genotype for South American strains (red, recognized as a-TC-Andean-Amazonian and a-TC-worldwide). The a-TC-Andean-Amazonian cluster is highlighted with a dark green circle, and a-TC-Worldwide cluster is highlighted with a dark brown circle. The a-TC-Worldwide includes 7 strains (IRID014, IRID034, IRID037, IRID057, IRID068, IRID080, and SS01) **E**) Geographic distribution of HTLV-1 clusters identified in Fig. 1D across South American samples, illustrating that the majority of samples from this region are of Andean-Amazonian origin. All statistical analyses and graphics were generated in RStudio using ggplot2

A total of 71 DNA samples—corresponding to 67 patients—were analyzed (including 4 patients with serial time-point samples). The median HTLV-1 genome coverage across all 67 patients in the Peruvian cohort was 158× (interquartile range [IQR]: 23–218×). After excluding 4 patients with less than 5× viral coverage (coincidentally, all with a PVL ≤ 0.25%), the final dataset comprised 63 cases (Supplementary Fig. 2A–C). In the final dataset, 3 cases (4.8%) harbored type II defective viruses (5’LTR-deleted; Fig. 1A). Notably, two of these cases developed ATLL and subsequently died, while the third died without apparent malignant transformation due to infectious complications during the COVID-19 pandemic. No type I defective viruses were detected in this cohort. The mean OCI for the entire cohort was 0.21 (95% CI: 0.17–0.26), and exhibited a low but significant correlation with PVL (Fig. 1B). No significant differences in OCI were observed regarding *Strongyloides* status (Table 1).

**Fig. 2:**
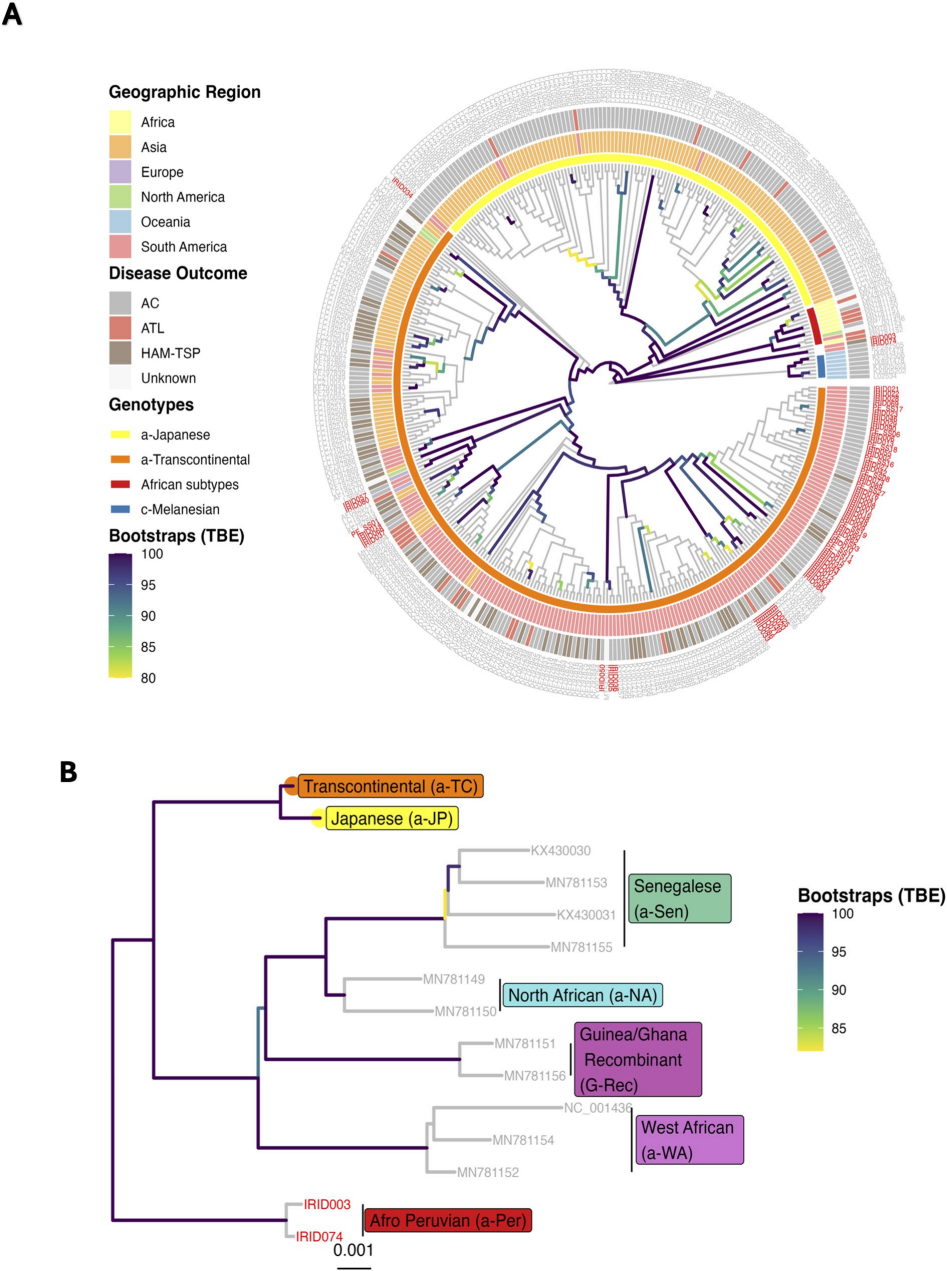
Genome-wide HTLV-1 global phylogenomic. **A**) A consensus phylogenetic tree was generated using RAxML, incorporating Transfer Bootstrap Expectation (TBE) support values. The tree achieved convergence after 5,000 bootstrap iterations using the GTR model (defined by ModelFinder) with three partitions: LTR, env, and the remainder of the genome. Bootstrap support values greater than 80 are displayed. The tree was rooted using the Australo-Melanesian clade (HTLV-1c) as the outgroup. New Peruvian strains are highlighted in red. Canonical classification based on LTR and env sequences is presented as solid bars adjacent to the tips. High genetic diversity is observed among Peruvian strains, with a dominant cohesive clade (a-TC-Andean-Amazonian) followed by geographically isolated strains of worldwide origin (a-TC-Worldwide: IRID057, IRID080, PE_SS01, IRID014, IRID068, IRID037). One isolated strain (IRID034) with low bootstrap support remained unclassified outside the TC clade, and two strains clustered with African subtypes (canonical a-Per). **B**) African subtypes are presented as a subset of Fig. 2A, with bootstrap support values greater than 80 and scaled evolutionary distances (branch lengths) displayed. Two new strains (IRID003 and IRID074) are highlighted in red and clustered outside both TC and African subtypes with high bootstrap confidence, assigned to the Afro-Peruvian subtype (also known as Black Peruvian; a-Per). These trees were generated in RStudio using ggtree and ggplot2

In the viral genomic analyses, 63 complete HTLV-1 genomes from Peru and 320 curated HTLV-1 genomes retrieved from NCBI were included (112 Brazilian, 1 Chilean, and 207 from other regions, Supplementary Fig. 3). After recombination screening using RDP4, SimPlot, and Bootscan, three sequences showed consistent recombination signals in at least three algorithms (KF797883, KY007245, KY007246, all of Brazilian origin) and were excluded from further phylogenetic analysis (Supplementary Table 1). Traditional genomic classification of the 63 Peruvian HTLV-1 isolates revealed that 61 (96.8%) belonged to the Cosmopolitan/Transcontinental (1a) subtype, while 2 (3.2%) were classified as African subtype (previously denominated as Black Peruvian) (Fig. 1C). Both African subtype cases originated from the coastal region of Peru, were of mestizo ethnicity, and one individual presented with HAM/TSP. The Andean-Amazonian (AA) subgroup was consistently identified as a distinct and well-defined clade within the Transcontinental cluster using unsupervised clustering algorithms (Fig. 1D-E). The maximum likelihood consensus tree is presented in Fig. 2A-B. The majority of South American samples clustered within the Transcontinental (subtype 1a) clade, primarily in the AA subgroup. However, 6 Peruvian cases (9.5%) fell outside the Andean-Amazonian subgroup, clustering instead with samples of worldwide origin, including isolates from Native Americans in Canada, Brazil, and Iran. A minority of Brazilian sequences (3.6%) clustered within the Japanese clade, potentially related to Japanese-descendant cases. One Peruvian sample (IRID034) showed direct phylogenetic relationship to the common Transcontinental ancestor, while two Peruvian isolates (IRID003 and IRID074, Fig. 2B) clustered separately from the Cosmopolitan subtype, showing closer genetic proximity to African subtypes. Under the prior assumption of African origin (early 1600 s) for these two samples outside the Transcontinental clade, the structured coalescent analysis revealed an ancient origin of the South American clade, with a divergence time estimation of 7,409 (95% CI: 5,064 − 9,020) years before present (Supplementary Fig. 4A-B). In this cohorts, most HTLV-1-related complications were observed in sequences from Transcontinental subtype viruses, with few HAM/TSP cases observed within the Japanese clade among publicly available complete HTLV-1 genomes.

### 3. HLA-I characteristics in the Peruvian HTLV-1 cohort

The Peruvian HTLV-1 cohort exhibited a unique HLA-I allele distribution, with A*02:01 being the most frequent allele at the HLA-A locus (45%), followed by A*24:02 (16%) and A*68:01 (7%). The HLA-B locus showed similar frequencies for B*35:01, B*35:05, and B*48:01 (10%, 11%, and 10% respectively). The HLA-C locus was dominated by C*04:01 (27%), followed by C*01:02 (19%) and C*07:02 (14%) (Supplementary Fig. 5A-B, Supplementary Tables 4–6). Notably, only one case exhibited potential loss of HLA heterogeneity (1.5%, IRID006) and coincidentally carried a HTLV-1 defective virus (5’-deletion). We performed HLA association analyses for mortality risk using a dominant inheritance model in the Peruvian cohort. The A03 supertype showed a trend toward higher crude mortality risk (OR: 5.73, 95% CI:1.34–29.96, p-value:0.02), while A02 supertype appeared protective (OR: 0.20, 95% CI: 0.04–0.95, p-value:0.04), though neither reached significance after correction (Supplementary Table 7, Supplementary Table 8). At allele nucleotide level, no significant associations were detected. At the amino acid level, several positions—mainly in HLA-A and HLA-C—showed signals, but none remained significant after Bonferroni correction (Supplementary Table 9). In addition, no associations were observed for HLA-I heterozygosity or HLA-NK ligands (Supplementary Table 10, Supplementary Table 11). In evolutionary terms, HLA-B locus divergence was associated with crude mortality risk, but this did not withstand multiple testing correction (Supplementary Table 12).

**Fig. 3:**
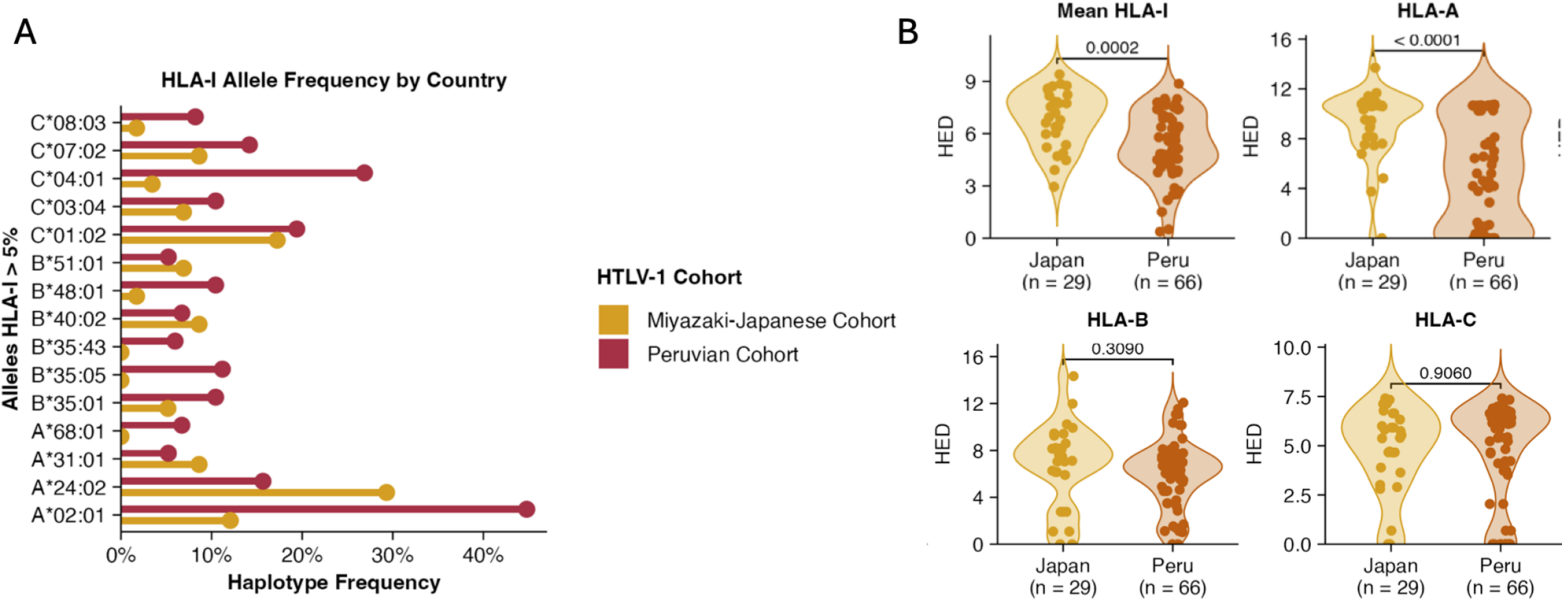
HLA-I diversity between HTLV-1 infected people from Japan and Peru. **A**) Allele frequencies for HLA class I alleles with prevalence greater than 5% are presented, derived from two independent genotyping algorithms (HLA-LA and Optitype). HLA-I allele frequency distributions were compared between the Peruvian HTLV-1 cohort (*n* = 66) and an independent HTLV-1/ATLL cohort from Miyazaki, Japan (*n* = 29). HLA-I allele frequencies were calculated in RStudio using the midasHLA package. **B**) HLA evolutionary distance (HLA-HED) for HLA-I locus-specific divergence was assessed using the Grantham distance metric, which accounts for amino acid composition, polarity, and molecular weight. HLA-HED was calculated using a custom Python script and compared as mean values across all HLA-I loci and independently for each individual locus. Statistical comparisons were performed using Welch’s t-test. Statistical analyses and visualizations were generated in RStudio using tidyverse, ggplot2, and tidyplots

Comparative analyses with a HTLV-1/ATLL cohort from Miyazaki (Japan) revealed population-specific structure (Fig. 3A). Hardy-Weinberg equilibrium was maintained for HLA-A (*p* = 0.11) and HLA-C (*p* = 0.25), while HLA-B showed significant deviation (*p* = 0.001), possibly reflecting HTLV-1 infection specific distribution among Peruvian cases. Pairwise F_ST_ analysis revealed significant genetic differentiation between the Peruvian and Japanese cohorts (mean F_ST_= 0.047, 95% CI: 0.021–0.079), confirming distinct HLA allele frequency distributions between these populations. We aimed to explore HLA-I evolutionary divergence (HLA-HED) between Miyazaki-Japanese and Peruvian HTLV-1 cohorts(Supplementary Fig. 5 C). We hypothesized that both populations exhibit differences in terms of diversity for peptide-binding(HLA-HED), which may explain differences in immune response between populations. Notably, the Peruvian cohort showed a reduced mean HLA-HED compared to the Japanese cohort(5.34 vs. 6.87, respectively; *p* = 0.0002), specifically this difference was mainly driven by HLA-A allele (Fig. 3B). Finally, we aimed to explore HLA-I evolutionary divergence (HLA-HED) regarding *Strongyloides* status (LSI vs. non-LSI), however, no significant differences were found in HLA-HED diversity (Supplementary Fig. 5D).

### 4. Clonal hematopoiesis in high-risk HTLV-1 carriers

We hypothesized that high-risk HTLV-1 carriers already harbor low-frequency mutations associated with ATLL development. To evaluate these early clonal lymphoid hematopoiesis events in high-risk HTLV-1 carriers, we performed ultra-deep targeted sequencing of 280 genes frequently mutated in hematological malignancies. Silent mutations were highly prevalent across the HTLV-1 cohort (Supplementary Fig. 6). *TRAF3* showed the highest recurrence rate at 98.5% of cases (median: 6 mutations per sample; IQR: 4–6), followed by *DUSP22* in 55.2% of cases (median: 3 mutations per sample; IQR: 2–4). The silent variations in *TRAF3* occurred predominantly in intronic regions (78.3%), with the most frequent variants being rs1274174947 (56% of cases) and rs180709957 (55% of cases). In contrast, silent variations in *DUSP22* showed a more balanced distribution across variant classifications, with rs55799519 (42% of cases) and rs3800260 (37% of cases) being the most prevalent variants. Analysis of the complete cohort revealed that the most recurrent polymorphisms were predominantly located in non-coding regions of *PRSS2*, *AKT2*, *TRAF3*, *CD24*, and *DUSP22* (Supplementary Table 13). These polymorphisms may represent important background genomic variation specific to HTLV-1-infected individuals, potentially influencing disease progression through non-coding regulatory mechanisms that modulate immune responses and viral control.

**Fig. 4:**
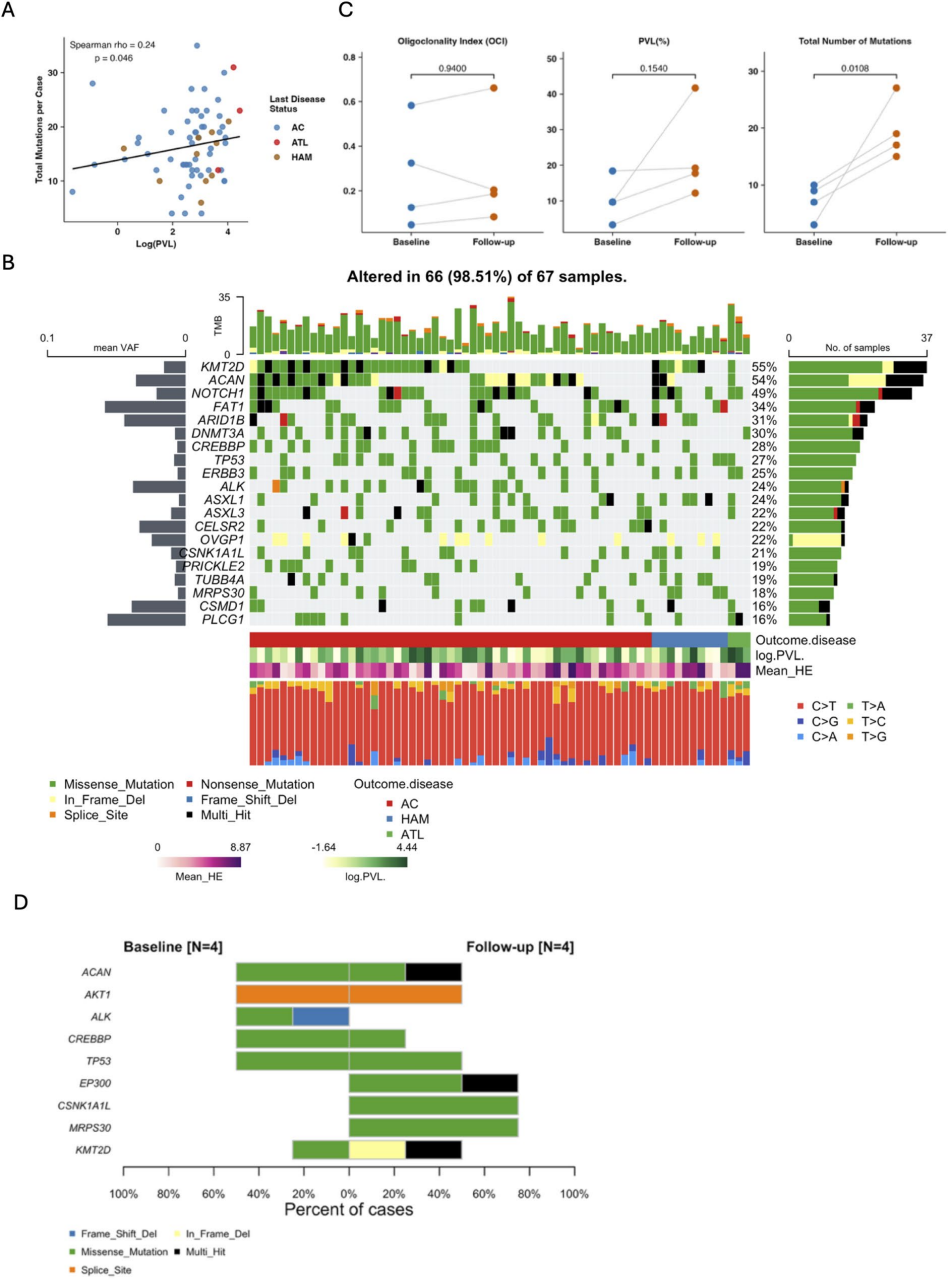
Clonal Hematopoiesis in HTLV-1-Infected Individuals. **A**) Correlation between proviral load and total somatic mutations per sample, correlation was calculated using Spearman Rho. Proviral load was logarithmically normalized before correlation. Total mutations per sample was obtained for every sample. Last outcome is shown in color red for ATLL, blue for AC, and brown for HAM/TSP B) Oncoplot displaying somatic mutations with a variant allele frequency (VAF) of at least 0.5%, excluding putative germline SNPs and synonymous variants (silent mutations). **B**) Correlation plot illustrating the relationship between the total number of mutations per sample and proviral load (PVL). **C**) Time-series evaluation of four representative samples, showing variations in OCI, PVL, and the number of mutations across multiple evaluations, paired-T test was performed to evaluate comparisons **D**) Oncobarplot comparing baseline and follow-up mutations, highlighting dynamic changes in mutational profiles over time. Plots were generated using Rstudio, maftools, ggplot2 and tidyplots

After excluding silent mutations (*n* = 4,691), we identified 1,101 non-synonymous mutations across all 67 samples, with a median of 15 mutations per case (IQR: 8–24). The mutation burden showed a significant positive correlation with HTLV-1 proviral load (Spearman *r* = 0.34, *p* = 0.003, Fig. 4A), suggesting that higher viral burden is associated with increased genomic instability. Missense mutations were the predominant variant type (87%), followed by in-frame deletions (5%) and splice site mutations (4%). The most frequently mutated genes were *KMT2D* (55%), *ACAN* (54%), *NOTCH1* (49%), *FAT1* (34%), *DNMT3A* (30%), *ARID1B* (31%), *CREBBP* (28%), and *TP53* (27%) (Fig. 4B, Supplementary Fig. 7, and Supplementary Fig. 8). In addition, significant pathways identified in the cohort were NOTCH (67%) and RTK-RAS (60%) pathways (Supplementary Fig. 9), while significantly mutated gene-based pathways involved histones modifiers (66%), as well as NOTCH (49%) and RTK (42%) signaling (Supplementary Fig. 10). *CSNK1A1* and *TP53* showed significantly co-occurring mutations, as did *MGAM* and *TUBB8*, while *OVGP1* and *ASXL1* exhibited mutually exclusive mutation patterns (Supplementary Fig. 11).

Finally, we evaluated PVL, OCI, and mutation burden in 4 representative samples with two serial collections to characterize the dynamics of clonal evolution over time (Fig. 4C and Supplementary Fig. 12). While PVL and OCI did not show significant changes between baseline and follow-up (3-years), the total number of mutations increased more than two-fold (7.25 vs. 19.5, *p* = 0.01). Interestingly, cumulative multi-hit mutations (e.g., *ACAN*, *EP300*, *KMT2D*) emerged during follow-up (Fig. 4D), while others became absent, suggesting progressive genomic instability and positive clonal selection during chronic HTLV-1 infection.

## Discussion

In the present study, we report the first comprehensive integrated analysis of viral and host genomics in HTLV-1 infection across the South American region. Using ultra-deep sequencing and high-resolution HLA typing, we evaluated a prospective Peruvian hospital-based cohort characterized by high-risk infection (median PVL 4.5%) and elevated crude mortality rate (13.4%), providing novel insights into the complex interplay between viral evolution, host genetics, and disease progression in an underrepresented population with the highest reported ATLL incidence globally [28]. 

The median PVL in this cohort was substantially higher than previously reported studies in other populations, including Australian Indigenous (< 1%) [29], Japanese (1.4%) [30], Brazilian (< 1% AC, ~ 20% HAM/TSP) [31], and Iranian cohorts (< 4%) [32], although similar to those found by another Peruvian group (11% AC, 26% HAM/TSP) [33]. While the underlying basis for this elevated PVL remains uncertain, several factors may contribute to this observation. First, the centralized hospital-based recruitment strategy likely introduced ascertainment bias toward more symptomatic cases [34], representing the “tip of the iceberg” of HTLV-1 infection burden in Peru. Second, population-specific genetic factors, including the reduced HLA evolutionary divergence may compromise viral control mechanisms compared to other populations. Specifically, the role of HLA functional diversity has been previously demonstrated in human immunodeficiency virus (HIV) infection, where greater functional divergence of HLA-A and HLA-B allotypes were associated with slower HIV progression by increasing peptide-binding repertoire diversity [35]. Similar mechanisms may operate in HTLV-1 infection. For instance, the HLA-I landscape in our Peruvian cohort differed markedly from previously studied populations with unique allele frequencies. Additionally, most previously described individual HLA-specific effects could not be replicated in this cohort [19], highlighting the critical need for population-specific HLA evaluations across diverse geographic and ethnic groups.

Importantly, we observed a strong correlation between low PVL and poor NGS coverage, suggesting that standard sequencing approaches may not be optimal for low-risk cases with minimal viral replication. High PVL was significantly associated with increased mortality risk (OR: 1.07; 95% CI: 1.01–1.15; *p* = 0.033), independent of ATLL development. This mortality association aligns with recent meta-analytical evidence [2], and suggest that elevated PVL represents a broader marker of immunological dysfunction and systemic inflammation rather than solely reflecting malignant transformation risk [15]. In addition, the use of a standardized percentage-based reporting system (with > 4% indicating high-risk infection) offers several advantages: (1) enhances clinical interpretability and health literacy among patients and providers, (2) facilitates meaningful comparisons across diverse populations and healthcare systems, and (3) provides a more intuitive framework for risk communication compared to traditional copy-number-based metrics [36]. 

Contrary to previous hypotheses suggesting *Strongyloides* infection as an ATLL prognostic factor [37], we could not confirm this association in our cohort. Our findings suggest that *Strongyloides* coinfection may represent a proxy marker or consequence of severe immunosuppression rather than directly fueling progression to ATLL. This interpretation is also supported by epidemiological data where *Strongyloides* prevalence was associated with elevated proviral loads [38]. Interestingly, HAM/TSP patients in our cohort exhibited lower rates of LSI compared to ACs, which may reflect distinct immunological profiles between these clinical states. In addition, the discovery of type II defective viruses, all associated with poor clinical outcomes, supports emerging evidence that defective proviruses may contribute to immune evasion and disease progression; however, their frequency was lower compared to a Japanese cohort (4.8% vs. 18.8%), and other defective subtypes were absent in this cohort [14]. 

Our phylogenomic analysis revealed the predominance of the Transcontinental (1a) subtype, with most isolates clustering within the newly proposed Andean-Amazonian subgroup (HTLV-1a-TC-Andean-Amazonian). This finding provides the molecular evidence for population-specific viral evolution in South America, with an estimated divergence time of 7,409 years before present. While the origin and introduction of HTLV-1 remains as a more philosophically controversial question [39–41], adequate priors based on ancient samples as well as more complete whole-genome sequenced isolates are required to increase credibility of Bayesian approaches. However, at least four distinct waves of HTLV-1 introduction into Peru can be described by phylogenomic analyses, highlighting the complex evolutionary history of the virus in this region.

Our mutation analysis provides the first evidence of early clonal hematopoiesis in high-risk HTLV-1 carriers, characterized by recurrent mutations in cancer-associated genes including *KMT2D* (55%), *NOTCH1* (49%), and *TP53* (27%). The positive correlation between mutation burden and proviral load (Spearman *r* = 0.34, *p* = 0.003), combined with the observed increase in mutation frequency during longitudinal follow-up, suggests that ongoing viral replication directly contributes to genomic instability in HTLV-1-infected cells. This finding supports a model whereby chronic viral activity promotes DNA damage and repair defects, leading to progressive accumulation of somatic mutations that may predispose to malignant transformation through positive clonal evolution. The high frequency of NOTCH (67%) and RTK-RAS (60%) pathway alterations indicates early dysregulation of key oncogenic pathways, potentially priming infected cells for malignant transformation. While most of these mutations have been reported in large-scale studies of Japanese and Western ATLL patients [20, 25, 42], these early clonal mutations appear to be implicated in the initial pathogenesis of HTLV-1-associated malignancies, particularly in Latin American populations. The prevalence of silent mutations in immune regulatory genes, particularly *TRAF3* (98.5% of cases) and *DUSP22* (55.2% of cases), represents an unexpected finding with potential mechanistic implications. These polymorphisms may modulate immune responses and viral control through non-coding regulatory mechanisms, contributing to the variable clinical outcomes observed in HTLV-1 infection.

### Study limitations

The hospital-based recruitment strategy may introduce selection bias toward cases with higher infection burden, potentially overestimating disease severity markers compared to population-based studies. The relatively small sample size limits statistical power for estimating time-to-event clinical outcomes and detecting rare genetic associations (HLA). Additionally, since most patients were recruited with high proviral load values, the rate of disease progression may be underestimated due to enrichment for already advanced cases. The lack of functional validation for identified mutations and HLA associations limits mechanistic interpretation of our findings, requiring future experimental studies to establish causality. Sequencing was performed on unsorted whole peripheral blood mononuclear cells rather than purified cell populations, which may obscure cell-type-specific mutational patterns and clonal dynamics. Finally, the limited longitudinal follow-up and absence of serial genomic assessments restricted our ability to fully characterize the temporal dynamics of clonal evolution and disease progression, preventing assessment of mutation acquisition patterns over time.

In conclusion, this comprehensive genomic characterization reveals population-specific features of HTLV-1 infection in Peru, including unique viral phylogeny, unique HLA-I diversity, and evidence of early clonal evolution. These findings provide a foundation to develop population-tailored and risk-based approaches for HTLV-1 management, and to highlight the critical need for expanded genomic surveillance for high-risk carriers in underrepresented populations bearing disproportionate infection burden. We emphasize the urgent need for investment in biobanking resources, establishment of prospective cohorts, and development of coordinated research strategies in Peru. Such efforts will be critical to advancing our understanding of HTLV-1 pathogenesis and improving outcomes for affected populations, as well as creating local capabilities through promoting local research and increasing awareness among vulnerable communities.

## Supplementary Information

Supplementary Material 1

## Data Availability

Data may be made available upon reasonable request by contacting the corresponding authors. Requests will be considered in accordance with ethical approval and applicable data protection regulations. All bioinformatics pipelines, custom scripts, and analytical workflows are publicly available in the project GitHub repository: https://github.com/deve105/htlv_paper_2025.
