# Supplementary material for: The viral and host genomic landscape of human T-cell leukemia virus type I in Peru
Source: Virol J. 2026 Apr 6;23:128. doi: 10.1186/s12985-026-03148-8 (PMC13191997; doi:10.1186/s12985-026-03148-8)
Supplement: Supplementary file 1 — Supplementary Material 1 [file 12985_2026_3148_MOESM1_ESM.pdf]

The Viral and Host Genomic Landscape of Human T-cell Leukemia Virus Type I in Peru

Daniel Enriquez-Vera, Jiazhou Li, Jorge Nakazaki-Aza, Kosuke Mochida, Yutaka Suzuki, Eduardo Gotuzzo, Martín Montes, Kazuhiro Morishita, Shingo Nakahata

Supplementary Methods Table 1~2

Supplementary Table 1~13

Supplementary Figure 1~12

Supplementary Methods Table 1. Software, versions and citations

Software	Citations
FASTQC (Version 0.12.1)	Andrews, S. (2010). FastQC: A Quality Control Tool for High Throughput Sequence Data [Online]. Available online at: http://www.bioinformatics.babraham.ac.uk/projects/fastqc/
Fastp (Version 0.23.4)	Shifu Chen. fastp 1.0: An ultra-fast all-round tool for FASTQ data quality control and preprocessing. iMeta 4.5 (2025): e70078
MultiQC (Version 1.27.1)	Ewels P, Magnusson M, Lundin S, Käller M. MultiQC: summarize analysis results for multiple tools and samples in a single report. Bioinformatics. 2016 Oct 1;32(19):3047–8.
BWA-MEM2 (Version 2.2.1)	Vasimuddin Md, Sanchit Misra, Heng Li, Srinivas Aluru. Efficient Architecture-Aware Acceleration of BWA-MEM for Multicore Systems. IEEE Parallel and Distributed Processing Symposium (IPDPS), 2019.
GATK (Version 4.3.0)	Van der Auwera GA & O'Connor BD. (2020). Genomics in the Cloud: Using Docker, GATK, and WDL in Terra (1st Edition). O'Reilly Media.
MAFFT (Version 7.49)	Katoh K, Standley DM. MAFFT multiple sequence alignment software version 7: improvements in performance and usability. Mol Biol Evol. 2013 Apr;30(4):772–80.
IQ-TREE (Version 3.0.1)	Nguyen LT, Schmidt HA, von Haeseler A, Minh BQ. IQ-TREE: A Fast and Effective Stochastic Algorithm for Estimating Maximum-Likelihood Phylogenies. Mol Biol Evol. 2015 Jan 1;32(1):268–74.
BEAST (Version 2.7)	Bouckaert R, Vaughan TG, Barido-Sottani J, Duchêne S, Fourment M, Gavryushkina A, et al. BEAST 2.5: An advanced software platform for Bayesian evolutionary analysis. PLOS Computational Biology. 2019 abr;15(4):e1006650.
RAxML-NG (1.2.2)	Kozlov AM, Darriba D, Flouri T, Morel B, Stamatakis A. RAxML-NG: a fast, scalable and user-friendly tool for maximum likelihood phylogenetic inference. Bioinformatics. 2019 Nov 1;35(21):4453–5.

Supplementary Methods Table 2. Model and Partition Selection

Partition Approach	Best Fit Model	Log-likelihood of consensus tree	AIC	AICc	BIC
Whole genome (unpartitioned)	TN+F+I+R3	-51,363.56	104,305.12	104,456.35	109,913.84
All regions included<br>Partition 1: LTR region, rest of genome, nucleotide position 2.<br>Partition 2:gag, pol-pro, env, pX, nucleotide position 1<br>Partition 3: nucleotide position 3	Partition 1: TIM2+F+I+R3<br>Partition 2: TN+F+I+R3<br>Partition 3: TN+F+R3	-92686.00	186,994.00	187,076.31	193,262.95
All regions included (and all genes with nucleotide position 1, 2, and 3)<br>Partition 1: LTR region, rest of genome, nucleotide position 2.<br>Partition 2: gag, pol-pro, env, pX, nucleotide position 1<br>Partition 3: nucleotide position 3	Partition 1: TIM2+F+I+R3<br>Partition 2: TN+F+I+R3<br>Partition 3: TIM2+F+I+R4	-92,986.88	187,593.62	187,676.77	193,893.50
Whole HTLV-1 genome and env (nucleotide position 3)<br>Partition 1: LTR region, rest of genome<br>Partition 2: gag, pol-pro, env, pX, env (nucleotide position 3)	Partition 1: TIM2+F+I+R3<br>Partition 2: TN+F+I+R3	-54,986.65	111,575.30	111,718.55	117,332.23
Whole HTLV-1 genome and env (nucleotide position 1, 2, and 3)<br>Partition 1: LTR region, rest of genome, env (nucleotide position 2)<br>Partition 2: gag, pol-pro, env, pX, env (nucleotide position 1 and 3)	Partition 1: TIM2+F+I+R3<br>Partition 2: TN+F+I+R3	-52667.42	106,936.83	107,088.38	112,652.46
LTR regions and genes with position 1, 2, and 3<br>Partition 1: LTR region, gag-env-pX (nucleotide position 2), pro-pol (nucleotide position 1), rest of genome<br>Partition 2: gag-env-pX (nucleotide position 1 and 3), pro-pol (nucleotide position 2 and 3)	Partition 1: TIM2+F+I+R3<br>Partition 2: TN+F+R3	-52,341.83	106,283.65	106,434.91	111,991.63
Paper's Model:<br>Partition 1: LTR region<br>Partition 2: env (nucleotide position 3)<br>Partition 3: rest of genome (gag, env/1-2, pX, pro-pol)	Partition 1: TIM2+F+R3<br>Partition 2: HKY+I+R2<br>Partition 3: TIM3+F+I+R3	-51,281.64	104,181.28	104,340.70	109,931.99

To determine the optimal evolutionary model for HTLV-1 phylogenomics, we compared multiple approaches: (1) whole-genome analysis using a single evolutionary model, and (2) partition-based analysis with partition-specific models. We extracted known gene annotations for HTLV-1 (reference sequence J02029.1) and systematically compared and merged partition schemes using

ModelFinder and PartitionFinder algorithms in IQ-TREE (version 2.3.5 for macOS ARM 64-bit). PartitionFinder identified three partitions optimized for model fit that align with HTLV-1 genomic organization: (1) LTR regions, (2) core genes (gag, pro, pol, env), and (3) auxiliary genes (px and HBZ). Model comparisons were based on AIC, AICc, and BIC scores. The three-partition model was selected as the best fit due to its significantly lower BIC score and superior log-likelihood compared to the whole-genome approach, indicating substantially improved model fit without overfitting.

Supplementary Table 1. Recombination Screening Across Multiple Algorithms

Recombinant Sequence(s)	Breakpoint Position Begin	Brreakpoint Position End	Minor Parental Sequence(s)	Major Parental Sequence(s)	RDP	GENECONV	Bootscan	Maxchi	Chimaera	SiSscan	PhylPro	LARD	3Seq
**KF797883.1***	8630	8740	L02534.1	KF797833.1	1.4E-46	3.44E-47	1.18E-39	9.91E-11	1.74E-10	1.54E-12	NS	3.89E-90	1.20E-40
**KY007245.1***	7358	7446	KX905202.1	KY007272.1	1.05E-09	0.45E-6	1.1E-9	NS	NS	NS	NS	8.85E-25	4.61E-02
**KY007246.1***	592	4576	KY007253.1	KY007262.1	2.87E-04	6.9E-2	2.9E-4	1.49E-05	4.53E-05	1.61E-05	NS	2.69E-4	2.47E-08
**A potential recombination signal was considered if at least four algorithms showed statistical significance, and such sequences were excluded from further analyses.<br>Analyses were performed using J02029.1 as the HTLV-1 reference genome in RDP (version 4.5) following default parameters.**													

Supplementary Table 2. Robust linear and beta regression model predicting HTLV-1 proviral load (PVL) based on demographic and clinical variables

	**Robust Linear Regression**			**Beta Regression**		
**Characteristic**	**Beta**	**95% CI**	**p-value**	**Beta**	**95% CI**	**p-value**
Age	0.04	-0.04, 0.11	ns	0.01	0.00, 0.02	0.2
Sex						
Female	—	—		—	—	
Male	-0.75	-2.7, 1.2	ns	-0.02	-0.37, 0.33	>0.9
LSI						
Strongyloides (-)	—	—		—	—	
Strongyloides (+)	1.4	-0.52, 3.4	ns	0.35	-0.02, 0.71	0.063
Birthplace						
Amazonian region	—	—		—	—	
Andean region	-1.7	-7.5, 4.0	ns	-0.51	-1.5, 0.47	0.3
Coastal region	-1.0	-6.5, 4.5	ns	-0.46	-1.4, 0.47	0.3

Abbreviation: CI = Confidence Interval; ns = non-significant; LSI: Lifetime Strongyloides Infection

Supplementary Table 3. Multivariable logistic regression model predicting mortality based on demographic, clinical, and proviral load variables

Characteristic	OR	95% CI	p-value
Age	1.02	0.93, 1.12	0.7
Sex			
Female	—	—	
Male	4.85	0.53, 90.6	0.2
Proviral Load	1.31	1.03, 1.79	0.047
LSI			
Strongyloides (-)	—	—	
Strongyloides (+)	0.44	0.04, 4.56	0.5

Abbreviations: CI = Confidence Interval, OR = Odds Ratio, LSI: Lifetime Strongyloides Infection

Supplementary Table 4. HLA allele frequencies in the Peruvian Cohort

allele	Peru_Cohort
A*02:01	0.447761194
A*02:06	0.007462687
A*02:07	
A*02:10	
A*02:105	0.014925373
A*02:11	0.029850746
A*02:121	
A*02:13	0.014925373
A*02:22	0.044776119
A*02:300	
A*02:319	
A*02:364	
A*02:64	0.007462687
A*02:73	
A*03:01	0.037313433
A*11:01	0.014925373
A*11:08	
A*11:32	
A*23:01	0.014925373
A*24:02	0.156716418
A*24:07	
A*24:310	
A*26:01	
A*26:03	
A*29:02	0.007462687
A*30:09	0.007462687
A*31:01	0.052238806
A*31:02	0.007462687

A*31:44	
A*33:01	0.007462687
A*33:03	
A*66:01	0.007462687
A*68:01	0.067164179
A*68:02	0.037313433
A*68:17	0.014925373
B*07:02	0.022388060
B*14:02	0.029850746
B*15:01	0.029850746
B*15:04	0.037313433
B*15:05	0.037313433
B*15:110	0.007462687
B*15:16	0.007462687
B*15:18	
B*15:32	
B*15:39	0.007462687
B*18:01	0.014925373
B*27:05	0.007462687
B*35:01	0.104477612
B*35:05	0.111940299
B*35:09	0.029850746
B*35:10	0.007462687
B*35:20	0.007462687
B*35:21	
B*35:22	0.007462687
B*35:43	0.059701493
B*39:01	0.014925373
B*39:03	0.014925373
B*39:05	0.029850746

B*39:06	0.014925373
B*39:09	0.022388060
B*39:12	0.014925373
B*39:13	0.007462687
B*39:18	
B*40:01	
B*40:02	0.067164179
B*40:04	0.014925373
B*40:06	
B*40:135	
B*40:211	
B*40:64	0.007462687
B*40:95	
B*44:02	0.007462687
B*44:03	0.022388060
B*45:01	0.007462687
B*46:01	
B*48:01	0.104477612
B*51:01	0.052238806
B*51:06	
B*51:75	0.007462687
B*52:01	0.007462687
B*53:01	0.007462687
B*54:01	
B*55:01	0.007462687
B*56:03	
B*57:01	0.014925373
B*58:01	0.007462687
B*59:01	
B*67:01	

C*01:02	0.194029851
C*03:02	
C*03:03	0.022388060
C*03:04	0.104477612
C*03:05	0.007462687
C*03:246	
C*03:38	
C*04:01	0.268656716
C*05:01	0.007462687
C*06:02	0.014925373
C*07:01	0.014925373
C*07:02	0.141791045
C*07:04	
C*07:242	
C*07:51	
C*07:96	
C*08:01	0.037313433
C*08:02	0.029850746
C*08:03	0.082089552
C*12:02	0.007462687
C*12:03	0.007462687
C*14:02	0.007462687
C*14:03	
C*14:28	
C*15:02	0.029850746
C*16:01	0.022388060
DPB1*01:01	0.029850746
DPB1*02:01	0.067164179
DPB1*03:01	0.044776119
DPB1*04:01	0.126865672

DPB1*04:02	0.402985075
DPB1*05:01	0.059701493
DPB1*09:01	
DPB1*100:01	
DPB1*13:01	0.052238806
DPB1*14:01	0.156716418
DPB1*17:01	0.022388060
DPB1*23:01	0.037313433
DPB1*269:01	
DQB1*02:01	0.044776119
DQB1*03:01	0.059701493
DQB1*03:02	0.037313433
DQB1*03:03	0.111940299
DQB1*03:113	
DQB1*04:01	
DQB1*04:02	0.119402985
DQB1*05:01	0.022388060
DQB1*05:02	
DQB1*05:03	0.022388060
DQB1*06:01	0.007462687
DQB1*06:02	0.014925373
DQB1*06:03	0.007462687
DQB1*06:04	0.014925373
DQB1*06:09	
DQB1*06:45	
DRB1*01:01	0.014925373
DRB1*01:02	0.022388060
DRB1*03:01	0.014925373
DRB1*03:02	0.007462687
DRB1*04:03	

DRB1*04:04	0.097014925
DRB1*04:05	
DRB1*04:06	
DRB1*04:07	0.126865672
DRB1*04:10	0.007462687
DRB1*04:11	0.022388060
DRB1*04:73	0.007462687
DRB1*07:01	0.022388060
DRB1*08:02	0.119402985
DRB1*08:03	
DRB1*09:01	0.246268657
DRB1*09:02	0.007462687
DRB1*11:01	
DRB1*11:04	0.022388060
DRB1*12:01	
DRB1*13:01	0.014925373
DRB1*13:02	0.014925373
DRB1*13:03	0.007462687
DRB1*14:01	0.014925373
DRB1*14:02	0.111940299
DRB1*14:03	
DRB1*14:05	
DRB1*14:06	0.007462687
DRB1*14:07	0.007462687
DRB1*15:01	0.022388060
DRB1*15:02	0.007462687
DRB1*15:03	0.007462687
DRB1*15:96	0.007462687
DRB1*16:02	0.014925373
DRB3*01:01	0.194029851

DRB3*02:01	0.014925373
DRB3*02:02	0.067164179
DRB3*03:01	0.037313433
DRB4*01:01	0.671641791
DRB4*03:01	0.283582090
E*01:01	0.641791045
E*01:03	0.298507463
F*01:01	1.000000000
G*01:01	0.723880597
G*01:03	0.067164179
G*01:04	0.119402985

Supplementary Table 5. Individual HLA-I genotyping

ID	A_1	A_2	B_1	B_2	C_1	C_2
IRID001	A*24:02:01	A*68:01:02	B*35:01:01	B*35:05:01	C*04:01:01	C*04:01:01
IRID002	A*02:01:01	A*03:01:01	B*07:02:01	B*35:01:01	C*04:01:01	C*07:02:01
IRID003	A*02:01:01	A*02:11:01	B*35:05:01	B*39:06:01	C*04:01:01	C*07:02:01
IRID005	A*02:01:01	A*68:01:02	B*15:05:01	B*40:02:01	C*01:02:01	C*03:04:01
IRID006	A*24:02:01	A*24:02:01	B*35:05:01	B*35:05:01	C*04:01:01	C*04:01:01
IRID007	A*68:01:02	A*68:01:02	B*35:09:01	B*51:01:01	C*01:02:01	C*15:02:01
IRID008	A*02:01:01	A*02:01:01	B*35:01:01	B*35:09:01	C*04:01:01	C*04:01:01
IRID009	A*23:01:01	A*24:02:01	B*40:64	B*58:01:01	C*03:04:01	C*07:01:01
IRID010	A*02:01:01	A*02:01:01	B*35:01:01	B*35:05:01	C*04:01:01	C*04:01:01
IRID011	A*02:01:01	A*68:02:01	B*15:16:01	B*57:01:01	C*06:02:01	C*14:02:01
IRID012	A*02:22:01	A*02:22:01	B*15:05:01	B*48:01:01	C*03:03:01	C*08:03:01
IRID013	A*02:01:01	A*68:17	B*35:20:01	B*48:01:01	C*04:01:01	C*08:01:01
IRID014	A*03:01:01	A*66:01:01	B*51:01:01	B*57:01:01	C*04:01:01	C*06:02:01
IRID016	A*02:01:01	A*24:02:01	B*15:01:01	B*15:110	C*01:02:01	C*01:02:01
IRID020	A*24:02:01	A*68:02:01	B*35:43:01	B*53:01:01	C*01:02:01	C*04:01:01
IRID021	A*02:01:01	A*02:01:01	B*35:05:01	B*39:01:01	C*04:01:01	C*07:02:01
IRID022	A*02:01:01	A*02:01:01	B*35:05:01	B*39:09:01	C*04:01:01	C*07:02:01
IRID023	A*02:01:01	A*24:02:01	B*35:43:01	B*39:13:01	C*01:02:01	C*07:02:01
IRID024	A*02:01:01	A*24:02:01	B*35:01:01	B*44:02:01	C*04:01:01	C*05:01:01
IRID025	A*02:01:01	A*02:22:01	B*35:43:01	B*39:12	C*01:02:01	C*07:02:01
IRID026	A*02:01:01	A*02:22:01	B*35:43:01	B*39:12	C*01:02:01	C*07:02:01
IRID027	A*02:01:01	A*31:02	B*35:05:01	B*39:05:01	C*04:01:01	C*07:02:01
IRID028	A*02:13	A*68:01:02	B*40:02:01	B*40:04	C*03:04:01	C*03:04:01
IRID029	A*02:01:01	A*02:01:01	B*44:03:01	B*48:01:01	C*08:03:01	C*16:01:01
IRID030	A*02:22:01	A*24:02:01	B*15:05:01	B*51:01:01	C*03:03:01	C*03:04:01
IRID032	A*02:01:01	A*68:02:01	B*14:02:01	B*35:05:01	C*08:02:01	C*08:03:01
IRID033	A*02:01:01	A*02:01:01	B*35:01:01	B*40:02:01	C*03:04:01	C*04:01:01
IRID034	A*02:01:01	A*24:02:01	B*35:05:01	B*40:02:01	C*03:05:01	C*04:01:01
IRID036	A*24:02:01	A*31:01:02	B*35:05:01	B*39:03	C*04:01:01	C*07:02:01
IRID037	A*02:01:01	A*31:01:02	B*35:01:01	B*35:05:01	C*04:01:01	C*04:01:01
IRID038	A*02:01:01	A*02:01:01	B*15:05:01	B*52:01:01	C*01:02:01	C*12:02:01
IRID040	A*30:09	A*31:01:02	B*15:01:01	B*39:05:01	C*01:02:01	C*07:02:01
IRID041	A*02:01:01	A*24:02:01	B*35:05:01	B*35:10	C*03:04:01	C*04:01:01
IRID042	A*02:01:01	A*02:01:01	B*39:09:01	B*48:01:01	C*07:02:01	C*08:03:01
IRID043	A*02:01:01	A*02:01:01	B*35:01:01	B*48:01:01	C*04:01:01	C*08:01:01

IRID046	A*02:01:01	A*02:11:01	B*07:02:01	B*51:01:01	C*07:02:01	C*08:01:01
IRID048	A*02:01:01	A*24:02:01	B*35:09:01	B*35:43:01	C*01:02:01	C*04:01:01
IRID049	A*02:01:01	A*11:01:01	B*27:05:02	B*35:09:01	C*01:02:01	C*04:01:01
IRID050	A*02:22:01	A*29:02:01	B*44:03:01	B*48:01:01	C*08:03:01	C*16:01:01
IRID055	A*02:01:01	A*02:11:01	B*39:05:01	B*51:01:01	C*07:02:01	C*15:02:01
IRID057	A*02:64:01	A*03:01:01	B*35:01:01	B*51:01:01	C*04:01:01	C*15:02:01
IRID060	A*02:01:01	A*68:01:02	B*15:04:01	B*39:01:01	C*01:02:01	C*07:02:01
IRID067	A*02:01:01	A*24:02:01	B*15:39:01	B*35:01:01	C*01:02:01	C*04:01:01
IRID068	A*02:01:01	A*02:01:01	B*15:04:01	B*35:01:01	C*01:02:01	C*01:02:01
IRID069	A*02:105	A*31:01:02	B*40:02:01	B*40:02:01	C*03:04:01	C*03:04:01
IRID071	A*02:01:01	A*24:02:01	B*39:09:01	B*48:01:01	C*07:02:01	C*08:03:01
IRID073	A*02:01:01	A*68:17	B*15:04:01	B*35:43:01	C*01:02:01	C*01:02:01
IRID074	A*02:01:01	A*24:02:01	B*15:01:01	B*40:04	C*01:02:01	C*03:04:01
IRID076	A*24:02:01	A*68:01:02	B*35:43:01	B*48:01:01	C*01:02:01	C*08:03:01
IRID080	A*02:01:01	A*02:01:01	B*48:01:01	B*51:01:01	C*03:04:01	C*08:03:01
IRID084	A*02:01:01	A*31:01:02	B*35:01:01	B*48:01:01	C*04:01:01	C*04:01:01
IRID086	A*33:01:01	A*68:01:02	B*14:02:01	B*39:06:01	C*07:02:01	C*08:02:01
IRID090	A*02:01:01	A*02:06:05	B*35:01:01	B*40:02:01	C*03:04:01	C*04:01:01
SS01	A*02:01:01	A*31:01:02	B*15:04:01	B*18:01:01	C*01:02:01	C*07:01:01
SS02	A*02:01:01	A*24:02:01	B*48:01:01	B*48:01:01	C*08:01:01	C*08:03:01
SS03	A*02:01:01	A*31:01:02	B*39:03	B*48:01:01	C*07:02:01	C*08:01:01
SS04	A*02:01:01	A*24:02:01	B*15:04:01	B*18:01:01	C*01:02:01	C*12:03:01
SS06	A*11:01:01	A*68:01:02	B*51:75	B*55:01:01	C*01:02:01	C*15:02:01
SS08	A*02:01:01	A*24:02:01	B*39:05:01	B*40:02:01	C*03:04:01	C*07:02:01
SS11	A*02:13	A*24:02:01	B*35:22	B*48:01:01	C*04:01:01	C*08:03:01
SS14	A*02:01:01	A*68:02:01	B*14:02:01	B*35:05:01	C*08:02:01	C*08:03:01
SS16	A*02:01:01	A*02:105	B*35:05:01	B*40:02:01	C*03:04:01	C*04:01:01
SS17	A*02:01:01	A*23:01:01	B*44:03:01	B*45:01:01	C*04:01:01	C*16:01:01
SS18	A*02:01:01	A*02:01:01	B*35:01:01	B*35:43:01	C*01:02:01	C*04:01:01
SS19	A*02:01:01	A*68:02:01	B*14:02:01	B*15:01:01	C*01:02:01	C*08:02:01
SS20	A*02:01:01	A*03:01:01	B*15:04:01	B*35:01:01	C*01:02:01	C*04:01:01
SS27	A*02:11:01	A*03:01:01	B*07:02:01	B*15:05:01	C*03:03:01	C*07:02:01

Supplementary Table 6. HLA allele frequencies compared to NMDP

allele	*Population*	*haplotype_freq*
A*02:01	HTLV-1 Peru	0.4444444444444440
A*02:01	Latino	0.2095
A*02:01	Japanese	0.148
A*02:01	AfricanAmerican	0.1235
A*24:02	HTLV-1 Peru	0.16666666666666700
A*24:02	Latino	0.1316
A*24:02	Japanese	0.353
A*24:02	AfricanAmerican	0.0245
A*31:01	HTLV-1 Peru	0.06349206349206350
A*31:01	Latino	0.0439
A*31:01	Japanese	0.0849
A*31:01	AfricanAmerican	0.01
A*68:01	HTLV-1 Peru	0.07142857142857140
A*68:01	Latino	0.048
A*68:01	Japanese	0.0017
A*68:01	AfricanAmerican	0.0395
B*35:01	HTLV-1 Peru	0.14285714285714300
B*35:01	Latino	0.0713
B*35:01	Japanese	0.0869
B*35:01	AfricanAmerican	0.0689
B*35:05	HTLV-1 Peru	0.09523809523809520
B*35:05	Latino	0.0056
B*35:05	Japanese	5E-04
B*35:05	AfricanAmerican	4E-04
B*40:02	HTLV-1 Peru	0.07142857142857140
B*40:02	Latino	0.0478
B*40:02	Japanese	0.0766
B*40:02	AfricanAmerican	0.0033
B*48:01	HTLV-1 Peru	0.1111111111111110
B*48:01	Latino	0.0142
B*48:01	Japanese	0.0271
B*48:01	AfricanAmerican	5E-04
B*51:01	HTLV-1 Peru	0.06349206349206350
B*51:01	Latino	0.0605
B*51:01	Japanese	0.089
B*51:01	AfricanAmerican	0.0217
C*01:02	HTLV-1 Peru	0.18253968253968300
C*01:02	Latino	0.0544
C*01:02	Japanese	0.1732
C*01:02	AfricanAmerican	0.0078
C*03:04	HTLV-1 Peru	0.1111111111111110
C*03:04	Latino	0.0602
C*03:04	Japanese	0.1269
C*03:04	AfricanAmerican	0.0565
C*04:01	HTLV-1 Peru	0.2857142857142860
C*04:01	Latino	0.1756
C*04:01	Japanese	0.0404
C*04:01	AfricanAmerican	0.2037
C*07:02	HTLV-1 Peru	0.1349206349206350

C*07:02	Latino	0.1214
C*07:02	Japanese	0.1218
C*07:02	AfricanAmerican	0.0713
C*08:03	HTLV-1 Peru	0.0873015873015873
C*08:03	Latino	0.0033
C*08:03	Japanese	0.0129
C*08:03	AfricanAmerican	7E-05

Supplementary Table 7. HLA-I allele associations with mortality risk under a dominant inheritance model

Allele	p-value	p-adjusted	estimate	std.error	conf.low	conf.high	statistic	N	%	Death	Death(%)
B*35:01	0.10	0.71	3.42	0.75	0.74	15.12	1.64	15	11.00%	11	22.00%
A*02:01	0.26	1.00	0.44	0.73	0.10	1.96	-1.13	48	36.00%	43	28.00%
C*04:01	0.49	1.00	1.65	0.72	0.40	7.27	0.69	30	22.00%	25	28.00%
C*01:02	0.49	1.00	1.64	0.73	0.37	6.91	0.68	23	17.00%	19	22.00%
C*07:02	0.66	1.00	0.69	0.85	0.10	3.21	-0.44	19	14.00%	17	11.00%
A*24:02	0.81	1.00	1.21	0.76	0.23	5.15	0.25	20	15.00%	17	17.00%
B*35:05	0.92	1.00	1.10	0.86	0.15	5.27	0.11	14	10.00%	12	11.00%

Supplementary Table 8. HLA-supertypes associations with mortality risk under a dominant inheritance model

Supertype	p-value	p-adjusted	estimate	std.error	conf.low	conf.high	statistic	N	%	Death	Death(%)
A03	0.02	0.23	5.73	0.77	1.34	29.96	2.27	21	16.00%	6	33.00%
A02	0.04	0.37	0.20	0.77	0.04	0.95	-2.09	55	41.00%	5	28.00%
B44	0.40	1.00	0.39	1.10	0.02	2.42	-0.85	15	11.00%	1	6.00%
B27	0.58	1.00	0.66	0.76	0.13	2.76	-0.55	28	21.00%	3	17.00%
B07	0.87	1.00	1.14	0.76	0.27	5.82	0.17	43	32.00%	6	33.00%
A24	0.89	1.00	1.11	0.76	0.22	4.73	0.14	21	16.00%	3	17.00%
B62	0.90	1.00	0.90	0.86	0.12	4.25	-0.13	16	12.00%	2	11.00%

Supplementary Table 9. HLA-I amino acid associations with mortality risk under a dominant inheritance model

Aminoacid	p-value	p-adjusted	estimate	std.error	conf.low	conf.high	statistic	N	%	Death	Death(%)
A_97_R	0.01	1	0.12	0.82	0.02	0.60	-2.61	58	43.00%	5	28.00%
A_9_F	0.02	1	0.14	0.80	0.03	0.71	-2.43	57	43.00%	5	28.00%
C_14_R	0.02	1	0.11	0.92	0.02	0.70	-2.40	61	46.00%	6	33.00%
C_49_A	0.02	1	0.11	0.92	0.02	0.70	-2.40	61	46.00%	6	33.00%
C_11_A	0.02	1	0.17	0.76	0.04	0.73	-2.37	52	39.00%	4	22.00%
A_62_G	0.02	1	0.17	0.78	0.04	0.83	-2.25	56	42.00%	5	28.00%
A_95_V	0.02	1	0.17	0.78	0.04	0.83	-2.25	56	42.00%	5	28.00%
A_107_W	0.02	1	0.17	0.78	0.04	0.83	-2.25	56	42.00%	5	28.00%
A_74_H	0.04	1	0.20	0.77	0.04	0.95	-2.09	55	41.00%	5	28.00%
A_116_D	0.04	1	4.82	0.76	1.14	25.01	2.06	23	17.00%	6	33.00%
B_114_N	0.05	1	0.23	0.74	0.05	0.99	-1.98	49	37.00%	4	22.00%

Supplementary Table 10. HLA-I heterozygozity associations with mortality risk

Aminoacid	p-value	p-adjusted	estimate	std.error	conf.low	conf.high	statistic	N	%	Death	Death(%)
C_het	0.16	0.47	0.32	0.80	0.07	1.75	-1.42	56	84.00%	6	67.00%
B_het	0.33	0.99	0.29	1.28	0.02	6.56	-0.98	64	96.00%	8	89.00%
A_het	0.99	1.00	1.01	0.86	0.21	7.34	0.01	52	78.00%	7	78.00%

Supplementary Table 11. HLA-NK-Ligands associations with mortality risk under a dominant inheritance model

Allele Group	p-value	p-adjusted	estimate	std.error	conf.low	conf.high	statistic	N	%	Death	Death(%)
C1	0.08	0.38	0.23	0.83	0.05	1.30	-1.77	58	43.00%	6	33.00%
C2	0.31	1.00	2.14	0.75	0.51	10.93	1.01	34	25.00%	6	33.00%
Bw4	0.36	1.00	0.50	0.75	0.10	2.09	-0.92	32	24.00%	3	17.00%
Bw4 (HLA-B only)	0.99	1.00	0.00	1,630.66		Inf	-0.01	16	12.00%	0	0.00%
Bw6	0.99	1.00	2,515,397.28	1,696.73	0.00		0.01	65	49.00%	9	50.00%

Supplementary Table 12. HLA-I divergence associations with mortality risk under a dominant inheritance model

HLA-I	p-value	p-adjusted	estimate	std.error	conf.low	conf.high	statistic
B	0.03	0.14	0.76	0.13	0.57	0.97	-2.11
ABC_avg	0.33	1.00	0.84	0.18	0.59	1.20	-0.98
C	0.60	1.00	0.93	0.14	0.72	1.24	-0.53
A	0.70	1.00	1.03	0.09	0.87	1.23	0.39

Supplementary Table 13. Recurrent silent mutations in HTLV-1 cohort (>10% frequency)

Gene Symbol	Genomic Position	Variant Type	Allele Change	Frequency (%)	Mean VAF	Population AF	dbSNP ID
PRSS2	chr7:142774515	DNP	TG>CA	74.6%	0.219	7.300	
PRSS2	chr7:142774511	SNP	C>T	71.6%	0.214	7.300	rs751504746
AKT2	chr19:40237754	DEL	AGCCACCACCCTGGACCTTGGTGGGGAGCCTGGTGAATGAGGGC>-	61.2%	0.011	1.960	rs1442987108
TRAF3	chr14:102877552	SNP	A>C	56.7%	0.065	1.400	rs1274174947
TRAF3	chr14:102876872	SNP	A>C	55.2%	0.036	1.850	rs180709957
CD24	chr6:106970848	SNP	T>C	44.8%	0.305	7.300	rs1426576929
DUSP22	chr6:320201	SNP	C>T	41.8%	0.267	1.010	rs55799519
MGAM	chr7:142059540	SNP	T>C	41.8%	0.264	0.507	rs2961074
MROH1	chr8:144251989	DEL	AGTGCCGGGTGCTGCTGCCCATCCTTTCTCCTTGCCGGGT>-	38.8%	0.006	7.300	
PLCG1	chr20:41174562	SNP	C>T	38.8%	0.006	7.300	
DUSP22	chr6:350371	SNP	C>T	37.3%	0.270	1.440	rs3800260
ENSG00000254192	chr5:168666079	SNP	A>G	37.3%	0.393	1.370	rs77328224
PRSS1	chr7:142750913	SNP	A>T	37.3%	0.100	7.300	rs745314232
PTENP1	chr9:33674779	SNP	T>A	37.3%	0.256	0.485	rs10814025
PTENP1	chr9:33674791	SNP	A>C	37.3%	0.273	0.485	rs10814026
ENSG00000273112	chr1:161594678	SNP	T>G	35.8%	0.547	0.452	rs77996283
ENSG00000290928	chr17:30760659	SNP	G>T	35.8%	0.267	0.943	rs11655677
PRSS2	chr7:142764019	SNP	C>T	35.8%	0.111	7.300	rs1231392174

Gene Symbol	Genomic Position	Variant Type	Allele Change	Frequency (%)	Mean VAF	Population AF	dbSNP ID
MGAM	chr7:142059474	SNP	T>C	34.3%	0.254	0.485	rs2960758
PRSS2	chr7:142764028	SNP	G>C	34.3%	0.121	7.300	rs796386253
IFNL2	chr19:39270107	SNP	G>A	32.8%	0.077	2.920	rs571836404
AKT1	chr14:104794093	SNP	C>T	31.3%	0.006	7.300	
CNKSR3	chr6:154388007	DEL	TTC>-	31.3%	0.861	0.583	rs142597714|rs564208311
FAS	chr10:89015534	SNP	C>T	31.3%	0.395	0.790	rs1468063
SASH1	chr6:148534963	DEL	GCTGCCAGTATGTGCTTAGGTCCCTGGAGTCACTTATTCTGTCCTGTTCTTTCTGTTACAAGAGTATGACAGTAAGTCCCTGTACGCACAGAGGTGTTCCCTGTGAGGTCTGCCACAGCAGGCCCCACCTAC>-	31.3%	0.433	7.300	
MROH1	chr8:144251948	DEL	GGTAGTGCCGGGTGCTGCTGCCCATCCTTCCTCTGCCGGGCAGTGCCGGGTGCTGCTGCCCATCCTTTCTCCTTGCCG>-	29.9%	0.004	7.300	
ARID1A	chr1:26774607	SNP	C>T	28.4%	0.006	7.300	
EPHB6	chr7:142867324	DEL	GTGTGGATGTGGGAGGGCTGTGGGCGTGTGTGTGTGTTGTGTGTCCCTGTGTGTGGATGTGGAGGGCTGTGGGGATGTGTGTGTGTGTTGTGTGTCCCTGT>-	28.4%	0.028	3.830	

Gene Symbol	Genomic Position	Variant Type	Allele Change	Frequency (%)	Mean VAF	Population AF	dbSNP ID
TRAF3	chr14:102877444	SNP	A>C	28.4%	0.037	2.000	rs1207843837
UBE2R2-AS1	chr9:33797339	SNP	G>A	26.9%	0.026	7.300	rs1163911938
ENSG00000271155	chr9:95507361	SNP	G>A	25.4%	0.008	4.610	rs1455303466
ENSG00000287265	chr6:291744	SNP	G>C	25.4%	0.329	0.408	rs9942546
HGH1	chr8:144138845	SNP	C>T	25.4%	0.006	7.300	rs768454477
LY9	chr1:160802289	SNP	C>T	25.4%	0.008	7.300	
SPEN	chr1:15937691	SNP	C>T	25.4%	0.008	7.300	
ACAN	chr15:88855498	SNP	A>G	23.9%	0.018	3.430	rs767359801
BIRC6	chr2:32618227	DEL	TA>-	23.9%	0.119	1.660	rs1491271030
CSMD1	chr8:3183288	SNP	T>C	23.9%	0.100	1.820	rs113529634
TRAF3	chr14:102876932	SNP	T>C	23.9%	0.027	2.680	rs1215966630
TRAF3	chr14:102876942	SNP	T>G	23.9%	0.017	2.550	rs1453473753
TRAF3	chr14:102905238	SNP	G>A	23.9%	0.006	7.300	
KPRP	chr1:152760104	SNP	C>T	22.4%	0.005	7.300	
LOX	chr5:122078664	SNP	A>G	22.4%	0.183	7.300	
STAT3	chr17:42313402	SNP	G>A	22.4%	0.114	1.240	rs200029754
TRAF3	chr14:102877491	SNP	A>G	22.4%	0.020	2.250	rs1173441728
CLCA1	chr1:86468932	SNP	G>A	20.9%	0.007	3.760	rs190336859
UBE2R2-AS1	chr9:33797326	DNP	CG>TA	20.9%	0.024	7.300	
ATP1A2	chr1:160142869	DNP	GC>TT	19.4%	0.457	7.300	rs886045432
CNKSR3	chr6:154395372	SNP	A>T	19.4%	0.419	1.050	rs35798842
TRAF3	chr14:102877231	SNP	C>T	19.4%	0.036	2.500	rs1317742234
ZNF142	chr2:218643537	SNP	C>T	19.4%	0.010	4.310	rs369246607

Gene Symbol	Genomic Position	Variant Type	Allele Change	Frequency (%)	Mean VAF	Population AF	dbSNP ID
ACAN	chr15:88855507	SNP	T>C	17.9%	0.010	3.490	rs62023517
ENSG00000198491	chr3:187200750	DEL	TG>-	17.9%	0.252	0.967	rs3086943|rs373077582
GPR39	chr2:132646321	SNP	G>A	17.9%	0.005	7.300	
IKZF1	chr7:50402440	SNP	G>T	17.9%	0.420	0.812	rs62447207
TNXB	chr6:32041186	SNP	C>T	17.9%	0.551	0.311	rs1058152
TRAF3	chr14:102877020	SNP	A>G	17.9%	0.022	2.400	rs143506170
ZNF286B	chr17:18672263	SNP	G>A	17.9%	0.007	7.300	
ARID1B	chr6:157150930	SNP	C>T	16.4%	0.005	7.300	
BCL6	chr3:187736667	SNP	G>A	16.4%	0.006	7.300	
ENSG00000264545	chr9:21971251	SNP	G>A	16.4%	0.007	4.610	rs1198823500
ENSG00000273112	chr1:161592632	SNP	A>G	16.4%	0.189	1.010	rs72700086
IL15	chr4:141716326	SNP	G>A	16.4%	0.005	7.300	
MGAM	chr7:142050332	SNP	C>A	16.4%	0.425	2.540	rs147875130
TRAF3	chr14:102876601	SNP	G>A	16.4%	0.007	3.100	rs549813533
TRAF3	chr14:102877681	SNP	T>C	16.4%	0.029	3.080	rs1485366547
TRAF3	chr14:102877753	SNP	A>G	16.4%	0.036	3.290	rs1432303851
ENSG00000273112	chr1:161594855	SNP	G>A	14.9%	0.014	2.870	rs1447247405
HNRNPA2B1	chr7:26198531	SNP	G>A	14.9%	0.009	7.300	
LRP1B	chr2:140232451	SNP	G>A	14.9%	0.006	7.300	
PTENP1	chr9:33676224	SNP	C>T	14.9%	0.007	7.300	
RECQL4	chr8:144513765	SNP	T>G	14.9%	0.062	0.845	rs35578669
SEC22B	chr1:120171845	SNP	C>T	14.9%	0.276	7.300	rs1267951541

Gene Symbol	Genomic Position	Variant Type	Allele Change	Frequency (%)	Mean VAF	Population AF	dbSNP ID
SUZ12	chr17:31976415	SNP	G>C	14.9%	0.021	2.300	rs867908533
TET2	chr4:105272773	SNP	C>T	14.9%	0.007	7.300	
ATP1A2	chr1:160142318	SNP	C>T	13.4%	0.005	7.300	
CADM1	chr11:115171316	SNP	C>T	13.4%	0.433	1.800	rs116941960
CYP21A2	chr6:32040013	SNP	C>G	13.4%	0.149	0.785	rs6477
DUSP22	chr6:325294	SNP	A>G	13.4%	0.037	2.070	rs28627213
ENSG00000230521	chr6:29887464	SNP	C>T	13.4%	0.314	1.040	rs116320566
ENSG00000290928	chr17:30759087	SNP	G>A	13.4%	0.316	0.914	rs55870272
FAT1	chr4:186619224	SNP	C>T	13.4%	0.005	7.300	
FAT1	chr4:186726506	SNP	G>C	13.4%	0.427	0.854	rs62342734
TAF2	chr8:119832683	SNP	C>T	13.4%	0.006	7.300	
TRAF3	chr14:102876704	SNP	A>G	13.4%	0.012	7.300	
TRAF3	chr14:102877793	SNP	C>T	13.4%	0.332	2.020	rs183026187
ATXN1	chr6:16753147	INS	->GG	11.9%	0.420	0.740	rs761477224
CDH9	chr5:26890549	SNP	C>T	11.9%	0.007	7.300	rs1460673875
CYP21A2	chr6:32039847	DNP	AC>GT	11.9%	0.342	7.300	rs71552100
DUSP22	chr6:326029	SNP	T>C	11.9%	0.029	7.300	rs1411871585
ENSG00000234211	chr1:161672560	SNP	C>T	11.9%	0.005	7.300	rs1323842438
ENSG00000253899	chr8:27065062	SNP	C>T	11.9%	0.011	7.300	rs962519076
FCGR2B	chr1:161677088	SNP	C>T	11.9%	0.006	4.130	rs757692645
HMGN2	chr1:26476294	SNP	C>T	11.9%	0.006	7.300	
HNRNPA2B1	chr7:26199860	SNP	G>A	11.9%	0.007	7.300	
JAK1	chr1:64867067	SNP	C>T	11.9%	0.007	7.300	rs1030379754

Gene Symbol	Genomic Position	Variant Type	Allele Change	Frequency (%)	Mean VAF	Population AF	dbSNP ID
KRAS	chr12:25207515	INS	->A	11.9%	0.887	0.058	rs56128001
TRAF3	chr14:102877054	SNP	A>C	11.9%	0.014	2.950	rs1162330003
ATXN1	chr6:16753143	DEL	AACA>-	10.4%	0.420	0.740	rs768741417
BCL2	chr18:63127889	SNP	G>A	10.4%	0.006	7.300	
CADM1	chr11:115172117	SNP	C>T	10.4%	0.435	0.381	rs11606837
ENSG00000273112	chr1:161600751	SNP	G>A	10.4%	0.241	1.080	rs143450775
FOXO3	chr6:108684385	SNP	A>G	10.4%	0.343	0.346	rs1062034
INTS1	chr7:1483418	SNP	C>T	10.4%	0.007	7.300	
INTS1	chr7:1502855	SNP	G>A	10.4%	0.009	7.300	rs768067796
IRS1	chr2:226732023	DNP	AC>TG	10.4%	0.615	7.300	rs386655835
PRSS2	chr7:142772607	SNP	A>G	10.4%	0.121	7.300	rs111256080
SUZ12	chr17:31976337	SNP	T>G	10.4%	0.021	2.340	rs541232464

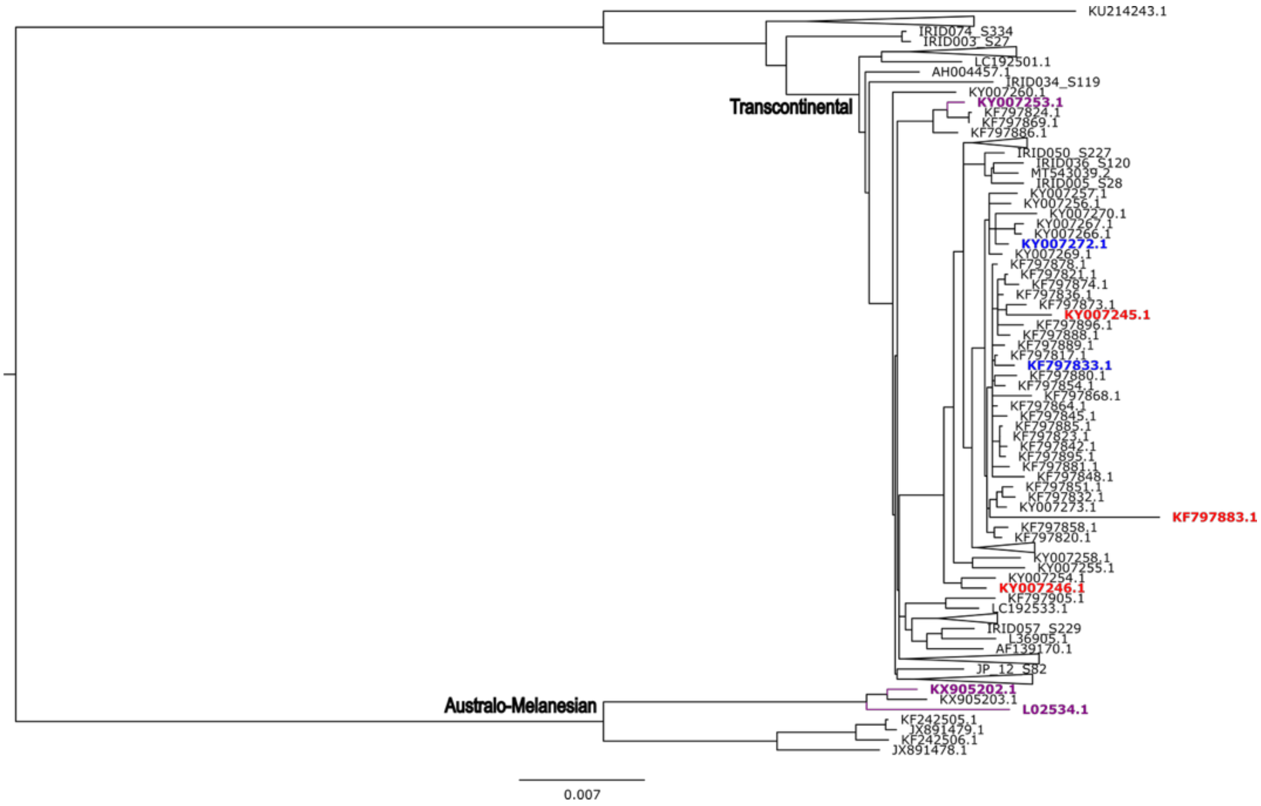

Supplementary Figure 1. Phylogenetic of HTLV-1 Strains with Potential Recombinant Signal. Maximum likelihood tree obtained in RAxML-NG with 5000 bootstraps using a complete genome-wide approach and the GTR evolutionary model, showing representative clades from the Transcontinental and Australomelanesian groups. The three sequences with potential recombinant signals (red: KF797883.1, KY007245.1, KY007246.1) display outlier distances relative to their reference clades (longer evolutionary distances). Major parents are indicated in blue (KY007272.1 and KF797833.1), and minor parents are indicated in violet (KX905202.1, KY007253.1, and L02534.1).

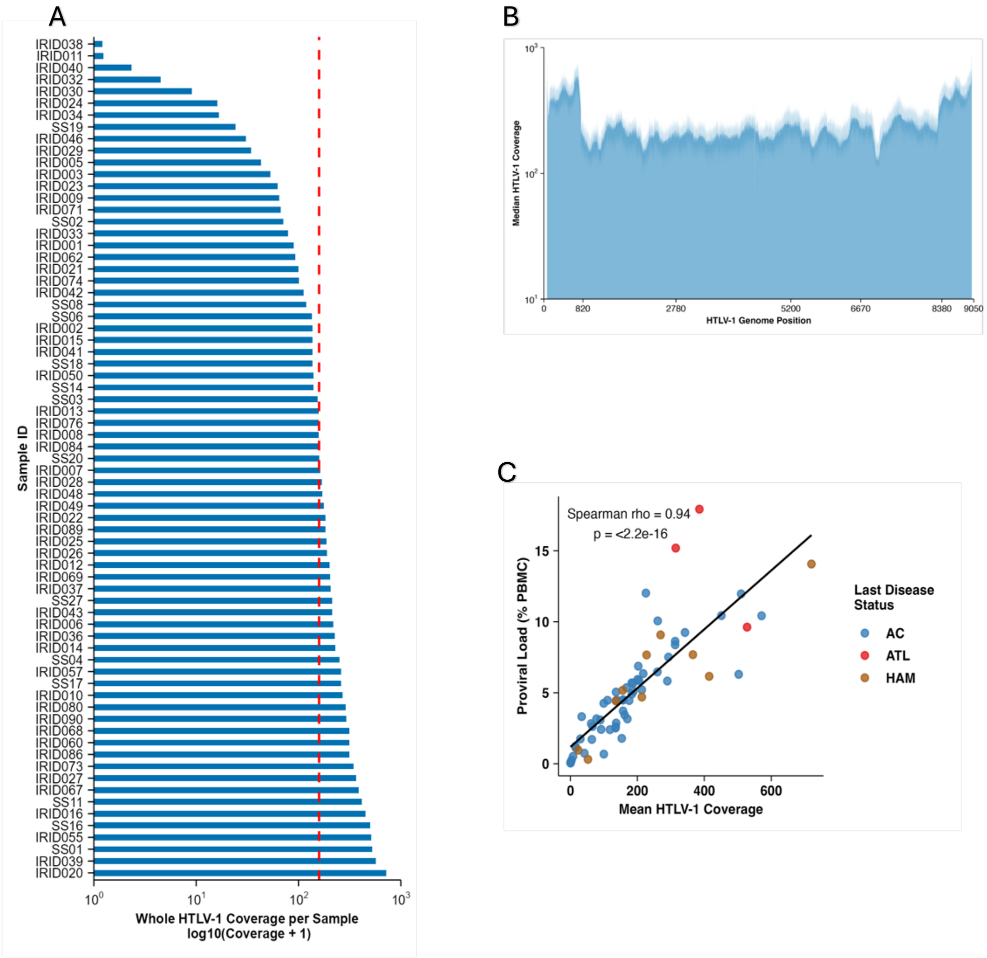

Supplementary Figure 2. A) Whole-genome HTLV-1 coverage per sample, expressed as log10-transformed values. The dashed red line represents the median coverage across all samples, highlighting variability in coverage levels. B) Median HTLV-1 coverage across the viral genome. C) Correlation between HTLV-1 genome coverage (Mean HTLV-1 Coverage) and Proviral Load (PVL), categorized by last disease status: AC (Asymptomatic Carriers), ATL (Adult T-cell Leukemia/Lymphoma), and HAM (HTLV-1-Associated Myelopathy/Tropical Spastic Paraparesis). The correlation was evaluated using Spearman's rho, with the black line representing the linear regression fit. This panel underscores the direct relationship between viral coverage and proviral load which justify the use of sequencing approaches in cases with high proviral load.

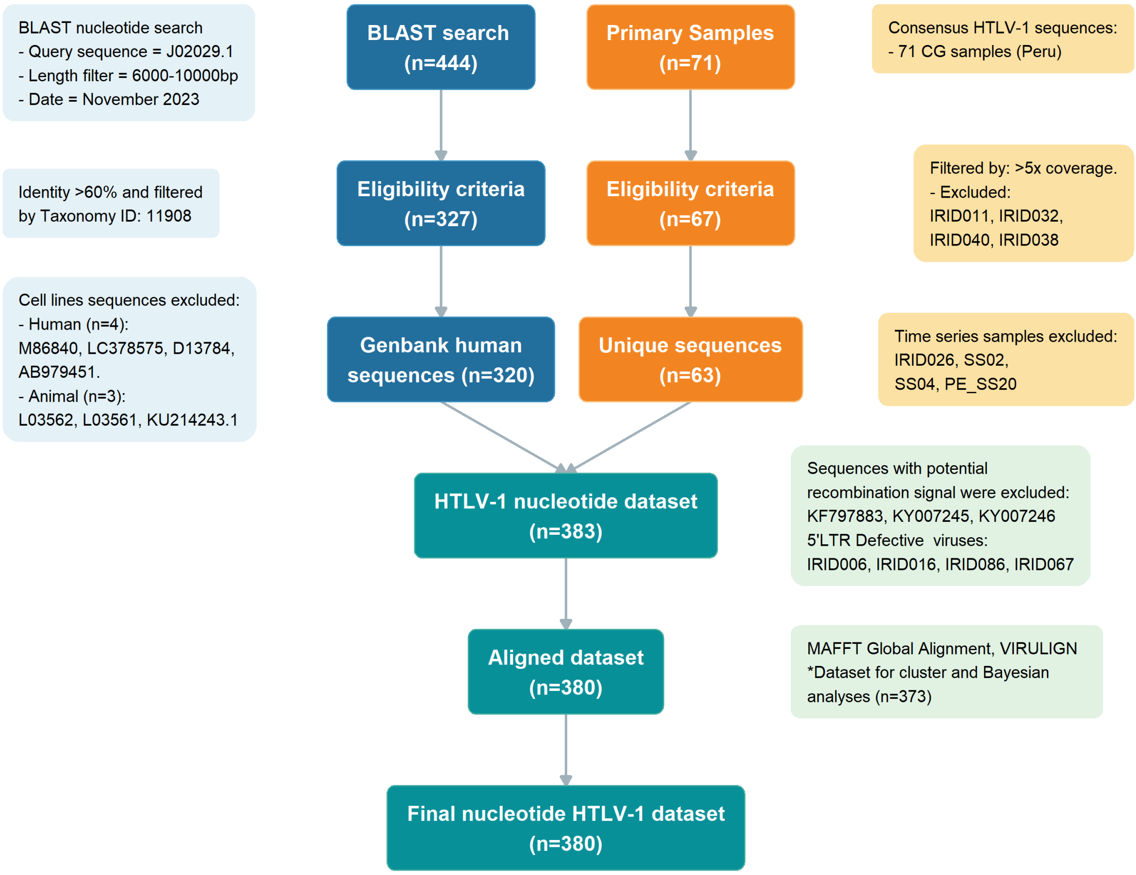

Supplementary Figure 3. Flowchart of sample filtering and final dataset preparation for HTLV-1 genomic alignment

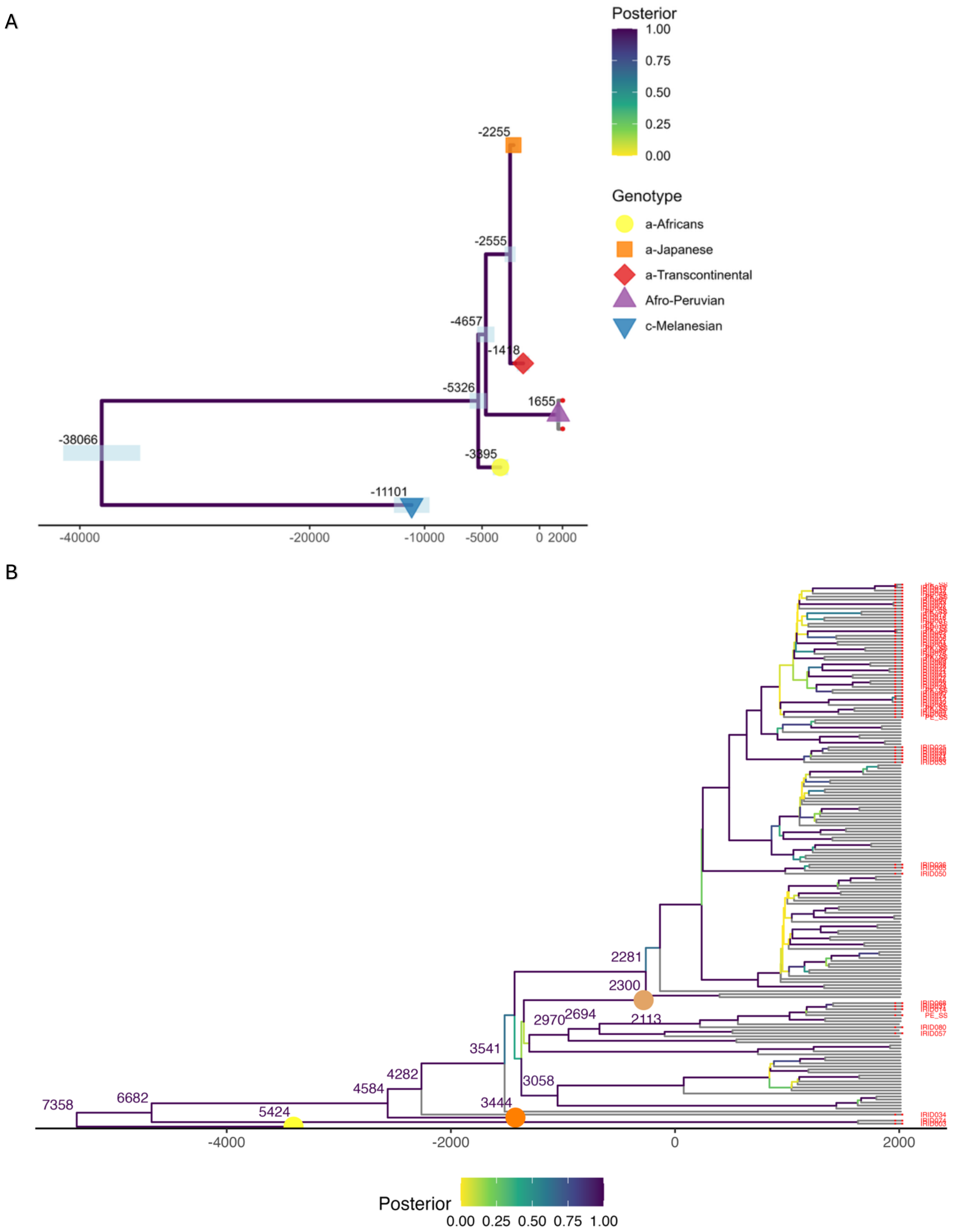
A
Posterior
1.00
0.75
0.50
0.25
0.00
Genotype
a-Africans
a-Japanese
a-Transcontinental
Afro-Peruvian
c-Melanesian
-2255
-2555
-4657
-1418
-5326
1655
-38066
-3395
-11101
-40000
-20000
-10000
-5000
0 2000
B
2281
2300
2970
2694
2113
3541
4282
4584
3058
3444
7358
6682
5424
-4000
-2000
0
2000
Posterior
0.00 0.25 0.50 0.75 1.00

Supplementary Figure 4. Bayesian phylogenetic tree of HTLV-1 complete genome sequences inferred using BEAST2 with a correlated relaxed clock model. Three partition-specific molecular clock priors were implemented to account for region-specific substitution rates: LTR (3.56×10^{-6} substitutions/site/year; 95% HPD: 5.4×10^{-7}–1.25×10^{-5}), env (7.3×10^{-6} substitutions/site/year; HPD: 1.73×10^{-6}–2.33×10^{-5}), and genome remainder (8.0×10^{-6} substitutions/site/year; HPD: 9.58×10^{-8}–1.9×10^{-5}). Substitution model priors were derived from maximum-likelihood model selection (IQ-TREE3) using GTR parameterization. A coalescent model with constant population size was employed as tree prior. Bayesian inference was performed using MCMC with 500 million iterations (sampling every 50,000), retaining 9,000 posterior samples after 10% burn-in. Convergence was confirmed by ESS > 200 for all parameters. Branch lengths represent mean divergence times (years before present); posterior probability support values (>0.95) are displayed at nodes. A) The collapsed tree includes representative sequences from all major HTLV-1 genotypes: Cosmopolitan subtypes [a-Jpn (Japanese), a-TC (Transcontinental which includes collapsed a-TC-Andean-Amazonian and a-TC-Worldwide], African subtypes (a-NA, a-WA, a-Sen, a-Rec, previously detailed in Figure 2B), and a-Per (Afro-Peruvian clade). Node age annotations facilitate identification of major evolutionary events and estimated timeframes for clade emergence and intercontinental dispersal patterns. Topology and divergence time estimates were validated through parallel maximum-likelihood inference (IQ-TREE3) using the identical partitioning scheme. B) Bayesian phylogenetic tree of HTLV-1 Cosmopolitan clade complete genome sequences inferred using BEAST2 with a correlated relaxed clock model, excluding HTLV-1c (Australo-Melanesian) outgroup and following the same priors of Supplementary Figure 4A. Analysis restricted to Cosmopolitan subtype including the Transcontinental (a-TC) clade, Japanese group (a-JPN in orange) and African subtypes (yellow). Branch lengths represent mean divergence times (internal nodes: mean years before present; YBP); posterior probability support values are displayed as branch colors. Subtypes are color-coded: dark orange represents a-Jpn (Japanese); lighter orange represents a-Jpn sequences nested within a-TC-Worldwide (Japanese strains with cosmopolitan distribution). Novel Peruvian HTLV-1 strains identified in this study are highlighted in red text.

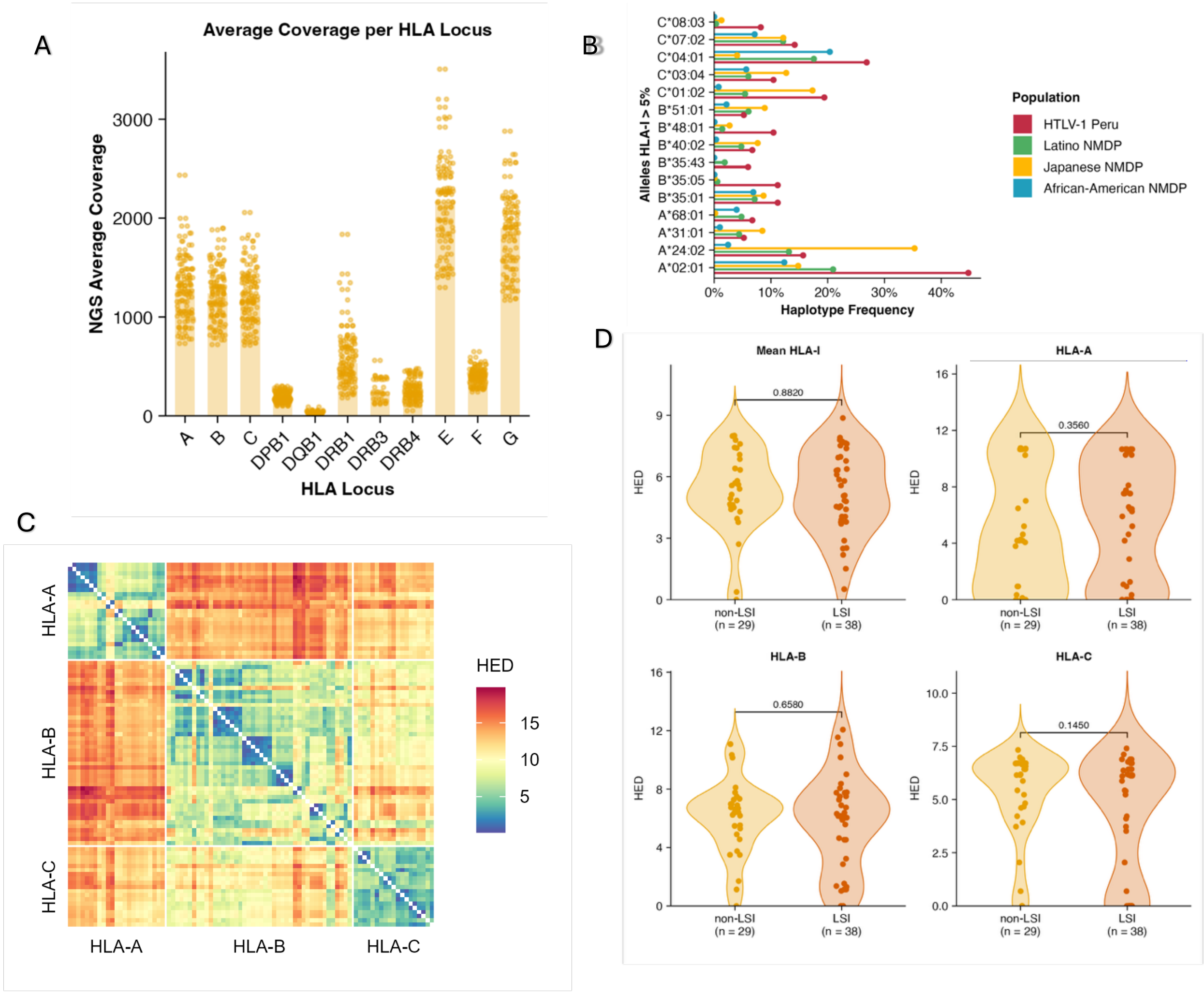

Supplementary Figure 5. A) Mean sequencing coverage depth across HLA class I genes obtained through next-generation sequencing (NGS). Coverage metrics were calculated per locus (HLA-A, HLA-B, HLA-C) to assess sequencing depth and genotyping confidence across the Peruvian HTLV-1 cohort (n=66). B) A) Allele frequencies for HLA class I alleles with prevalence >5% are presented, derived from two independent genotyping algorithms (HLA-LA and Optitype) to ensure genotyping accuracy and reproducibility. HLA-I allele frequency distributions were compared between the Peruvian HTLV-1 cohort (n=66) and reference populations from the National Marrow Donor Program (NMDP) database, including Latino, Japanese, and African American individuals. Comparative analysis of allele frequencies across these geographically and ethnically distinct populations provides context for identifying population-specific HLA genetic architecture and potential associations with HTLV-1 disease susceptibility or progression. C) Heatmap displaying pairwise HLA evolutionary distances (HLA-HED) among HLA class I alleles, calculated using Grantham distance metric, which accounts for amino acid composition, polarity, and molecular weight. Hierarchical clustering based on HED values reveals allelic relationships and identifies

allele groups with similar evolutionary properties. D) Comparison of mean HLA-HED scores (HLA diversity index) between non-LSI (low-burden HTLV-1 individuals; n=29) and LSI (high-burden HTLV-1 individuals; n=38) groups. Statistical comparison was performed using Welch's t-test; no statistically significant difference was observed between groups (p=0.88). HLA-HED was compared both as aggregate value across all HLA-I loci and independently per locus. All analyses were performed in RStudio using custom R scripts, tidyverse, ggplot2, pheatmap, and tidyplots.

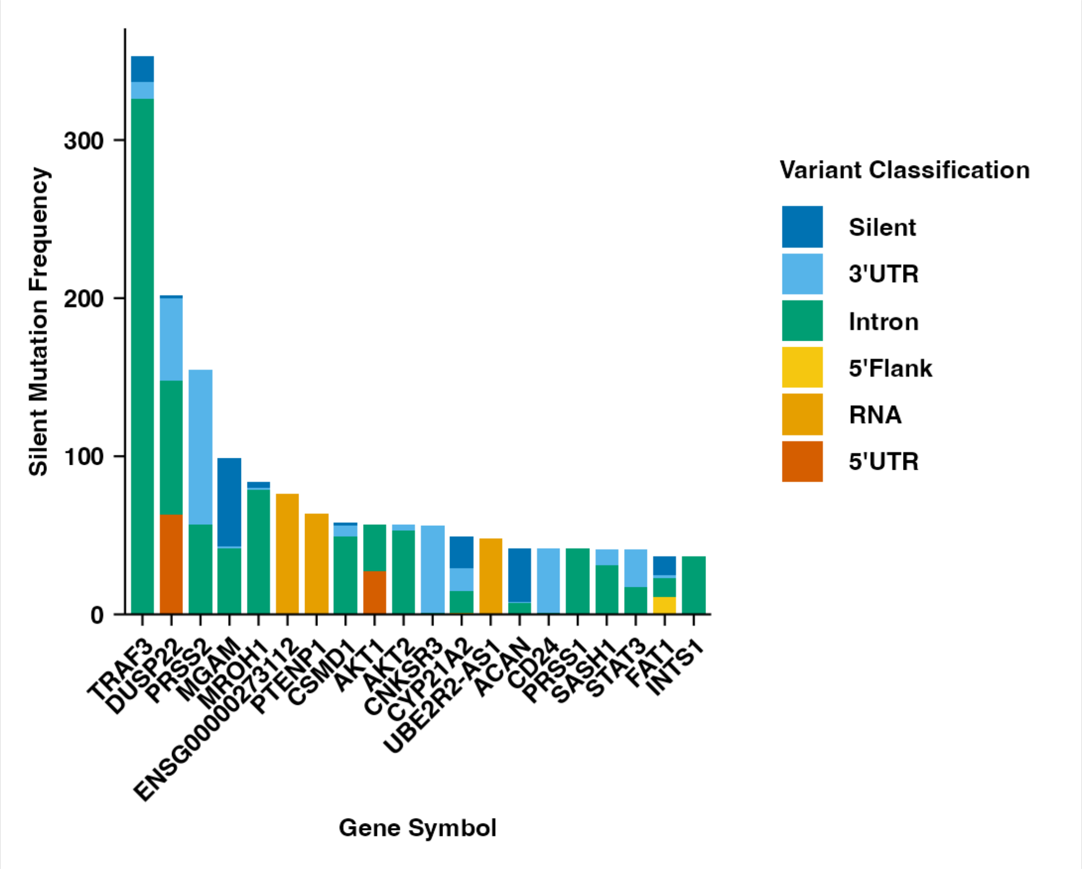

Supplementary Figure 6. Summary of somatic mutations obtained from the HTLV-1 cohort. Summary plot includes somatic variant classification, variant type, SNV subtype, number of variants per sample, and top 20 mutated genes in the entire cohort. Somatic SNV were obtained using Mutect2 pipeline and Funcotator and presented using Oncoplot.

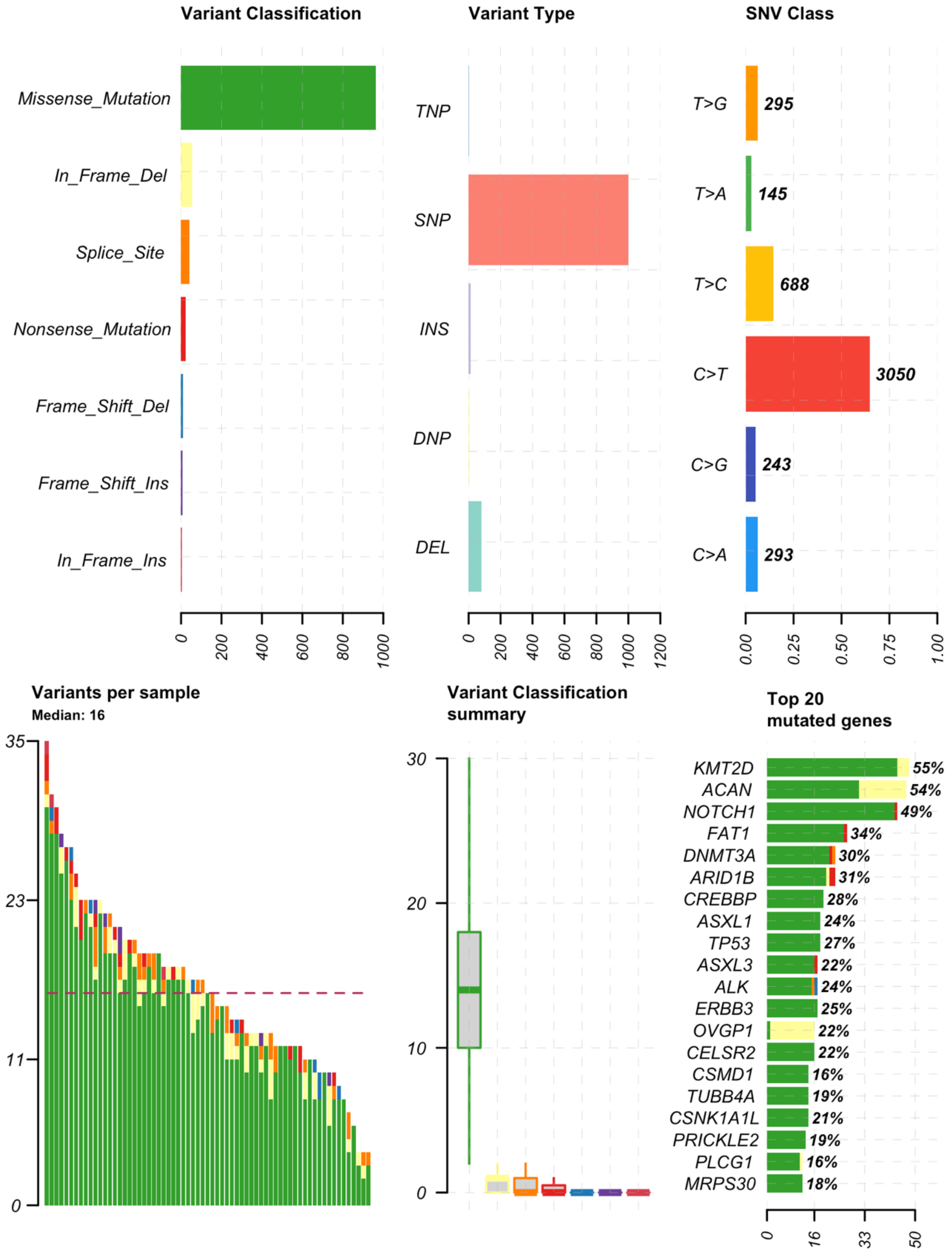

Supplementary Figure 7. Summary of somatic mutations obtained from the HTLV-1 cohort. Summary plot includes somatic variant classification, variant type, SNV subtype, number of variants per sample, and top 20 mutated genes in the entire cohort. Somatic SNV were obtained using Mutect2 pipeline and Funcotator and presented using Oncoplot.

KMT2D : [Somatic Mutation Rate: 55.22%]
NM_003482

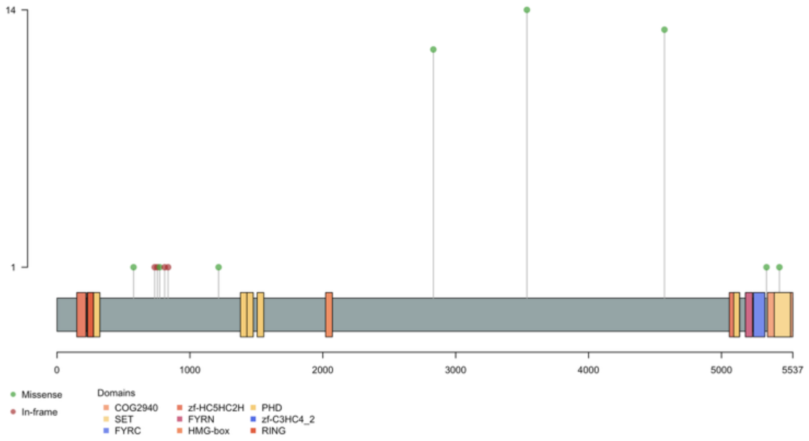

ACAN : [Somatic Mutation Rate: 53.73%]
NM_013227

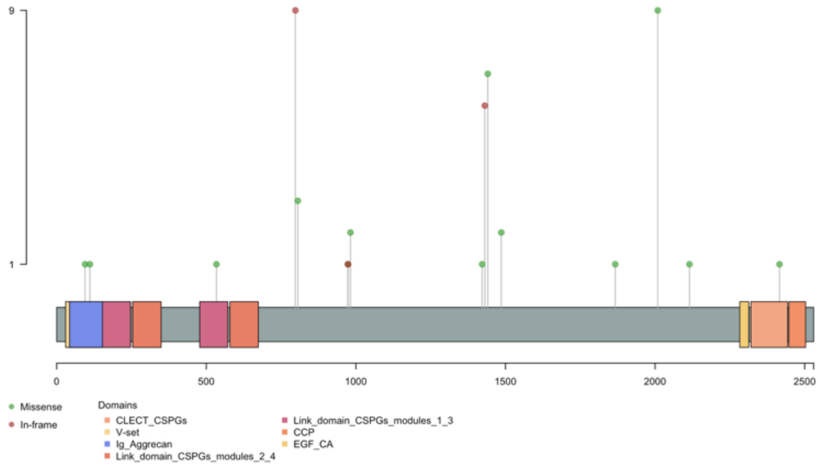

NOTCH1 : [Somatic Mutation Rate: 49.25%]
NM_017617

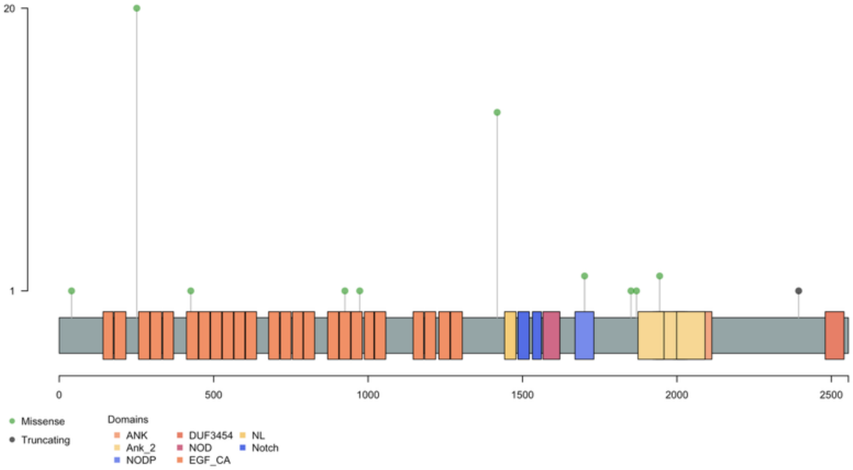

Supplementary Figure 8. Lollipop plot of A) KMT2D, B) NOTCH1, and C) ACAN recurrent mutations localizations across the HTLV-1 Peruvian cohort (n=67). Plots were generated in Rstudio using maftools.

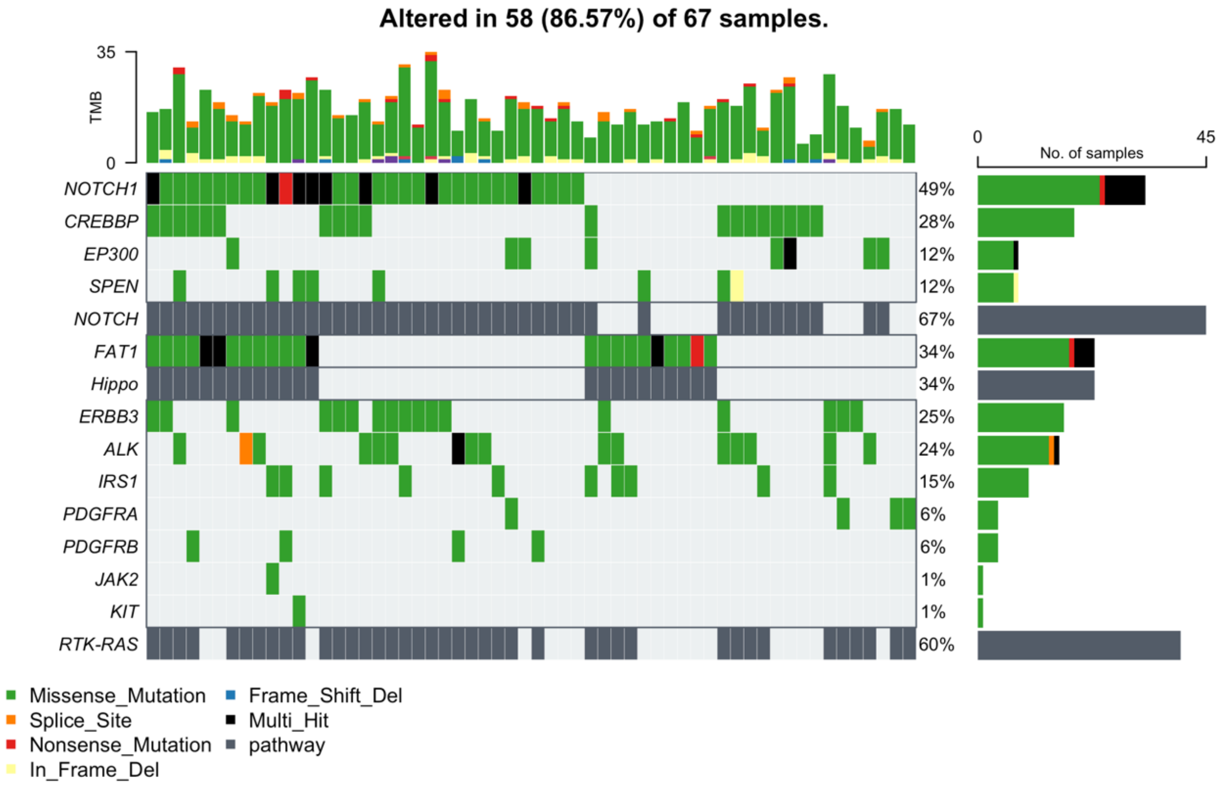

Supplementary Figure 9. Significant pathways identified in the cohort

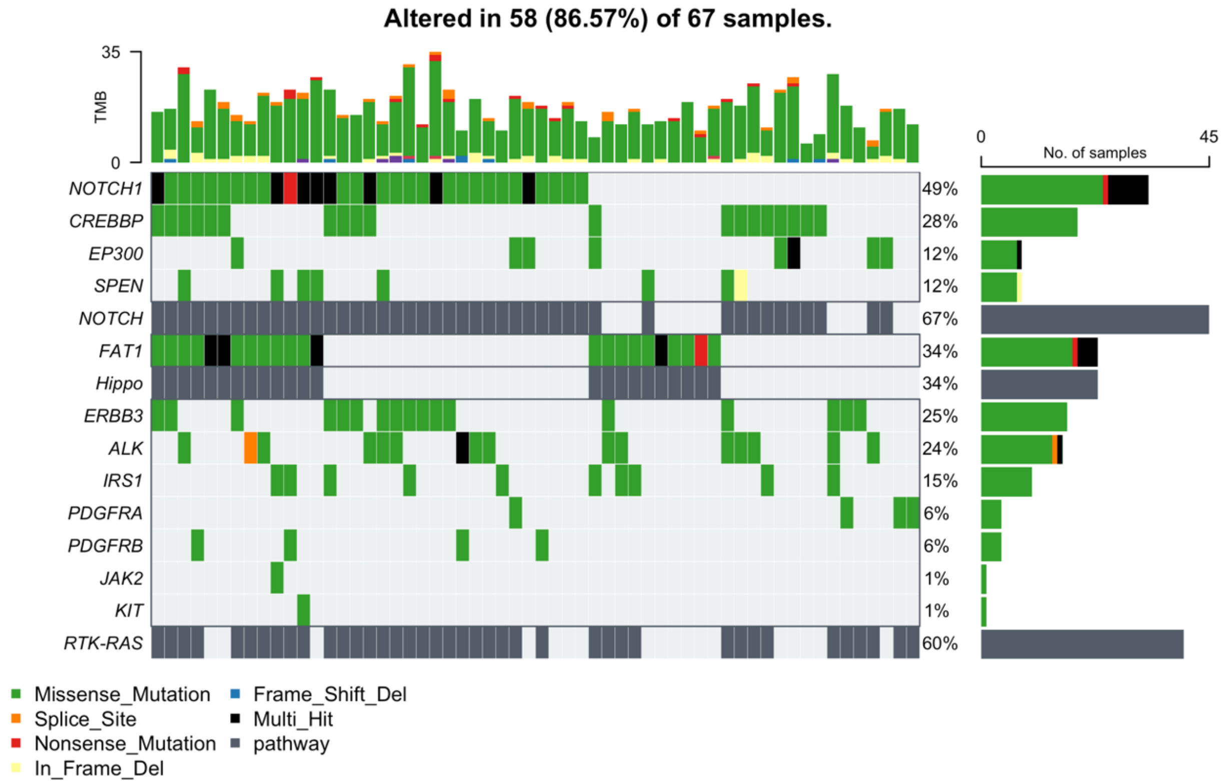

Supplementary Figure 10. Signifcantly mutated gene-based pathways

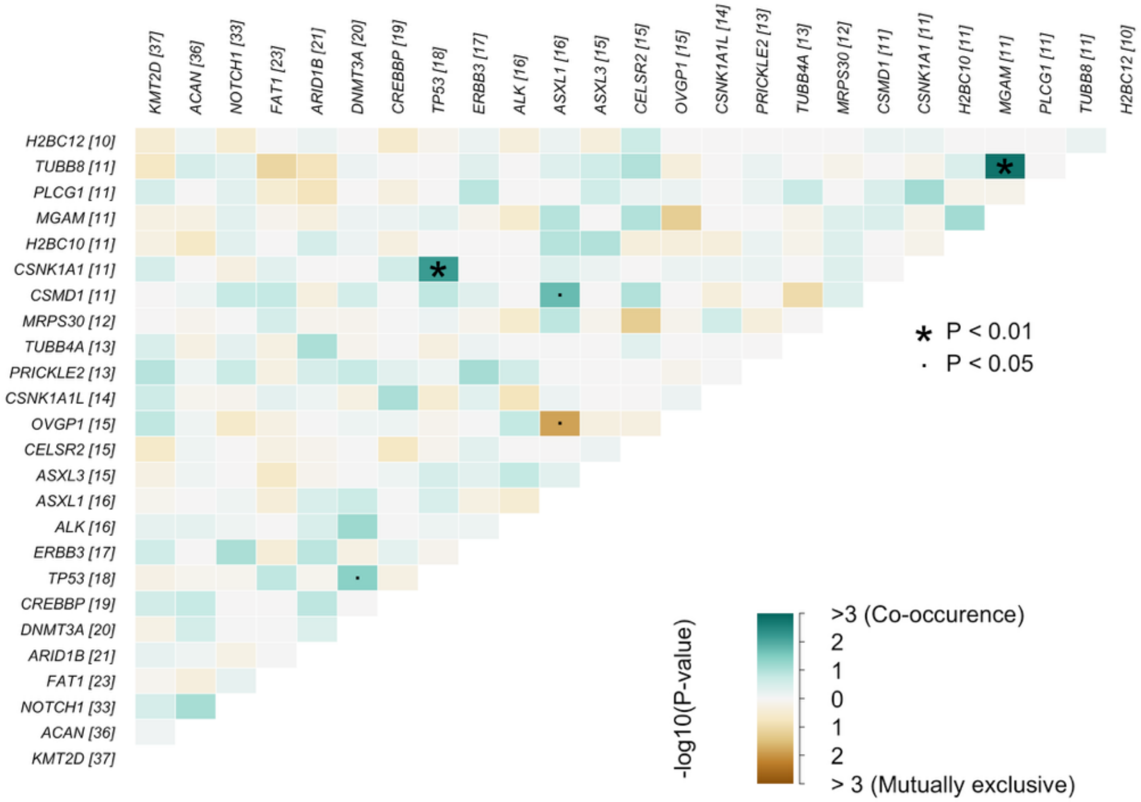

Supplementary Figure 11. Co-ocurrence analysis of somatic mutations

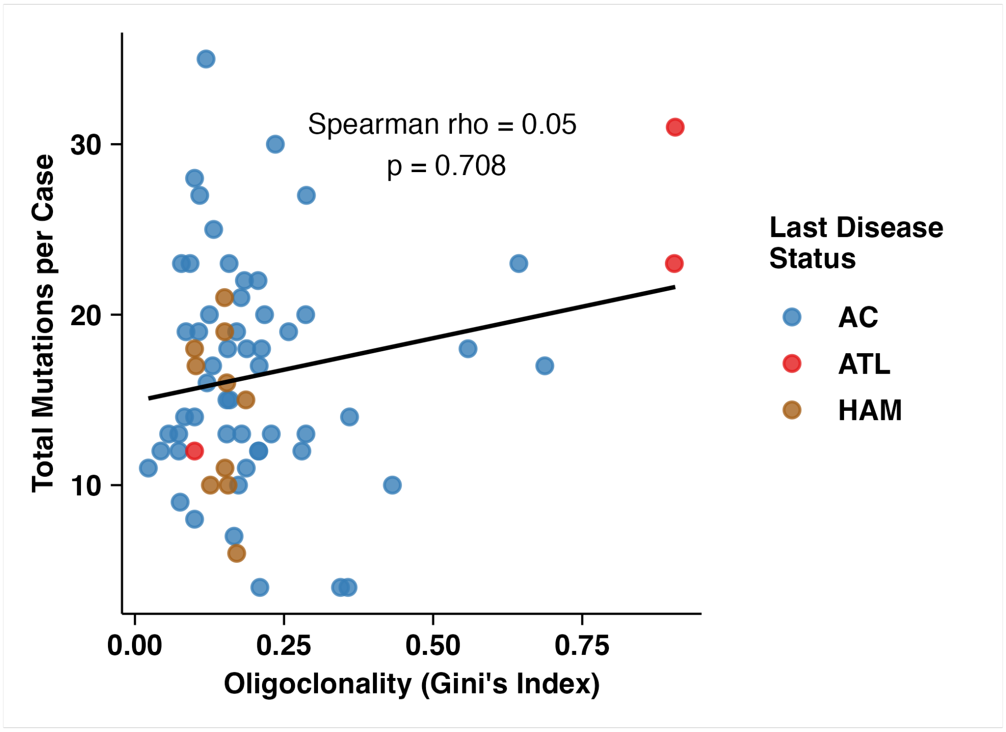

Supplementary Figure 12. Correlation between total mutations per case and oligoclonality (Gini's index). (Gini's index). Clinical outcomes are color-coded: AC (Asymptomatic Carriers, blue) and ATLL (Adult T-cell Leukemia/Lymphoma, red). Spearman rank correlation analysis revealed no statistically significant association between oligoclonality and mutation burden (Spearman $\rho = 0.05$, $p = 0.71$). The mutational burden analysis was based exclusively on non-synonymous mutations with variant allele frequencies (VAF) between 0.5% and 30% to exclude potential germline alterations, following stringent quality criteria: minimum coverage of 500×, at least 5 high-quality paired-end reads, and additional strict filters detailed in Supplementary Methods 5. Despite substantial proviral burden across the cohort, the median oligoclonality index was notably low, with only four cases exceeding 0.6, which may underestimate the true biological impact of clonal selection on HTLV-1 persistence. The limited number of oligoclonal events (n=4) substantially constrains the statistical power for accurate estimation of mutational signatures and clonal architecture.
